# Vertical sleeve gastrectomy reverses diet-induced gene-regulatory changes impacting lipid metabolism

**DOI:** 10.1038/s41598-017-05349-2

**Published:** 2017-07-13

**Authors:** Juan Du, Jingyan Tian, Lili Ding, Candi Trac, Brian Xia, Siming Sun, Dustin E. Schones, Wendong Huang

**Affiliations:** 1 Department of Diabetes Complications and Metabolism, Beckman Research Institute, City of Hope, Duarte, CA USA; 2 Irell & Manella Graduate School of Biological Sciences, City of Hope, Duarte, CA USA; 3 Shanghai Clinical Center for Endocrine and Metabolic Diseases, Shanghai Institute of Endocrine and Metabolic Diseases, Department of Endocrinology and Metabolism, China National Research Center for Metabolic Diseases, Ruijin Hospital, Shanghai Jiao Tong University School of Medicine, Shanghai, China

## Abstract

Vertical sleeve gastrectomy (VSG) produces sustainable weight loss, remission of type 2 diabetes (T2D), and improvement of nonalcoholic fatty liver disease (NAFLD). However, the molecular mechanisms underlying the metabolic benefits of VSG have remained elusive. According to our previous results, diet-induced obesity induces epigenetic modifications to chromatin in mouse liver. We demonstrate here that VSG in C57BL/6J wild-type male mice can reverse these chromatin modifications and thereby impact the expression of key metabolic genes. Genes involved in lipid metabolism, especially omega-6 fatty acid metabolism, are up-regulated in livers of mice after VSG while genes in inflammatory pathways are down-regulated after VSG. Consistent with gene expression changes, regulatory regions near genes involved in inflammatory response displayed decreased chromatin accessibility after VSG. Our results indicate that VSG induces global regulatory changes that impact hepatic inflammatory and lipid metabolic pathways, providing new insight into the mechanisms underlying the beneficial metabolic effects induced by VSG.

## Introduction

A dramatic increase in the prevalence of obesity over the last few decades has led to a worldwide epidemic. In the United States alone, one-third of adults are classified as obese (BMI > 30)^1^. Obesity is a risk factor for many other diseases, including type 2 diabetes (T2D), cardiovascular diseases, and many types of cancer^2^. Conventional treatments for obesity such as exercise and dietary changes are often insufficient leading to regaining weight. Pharmacological options are limited and patients often experience severe side effects^3^. Bariatric surgeries, including vertical sleeve gastrectomy (VSG), Roux-en-Y gastric bypass and adjustable gastric band, have shown sustainable weight loss and remission of T2D^4^. VSG is not only an effective treatment for obesity and T2D, but also has been shown to significantly improve nonalcoholic fatty liver disease (NAFLD) and nonalcoholic steatohepatitis (NASH)^5, 6^. Obese mice that receive VSG have reduced hepatic steatosis *prior to* weight loss^5^. Despite these benefits, bariatric surgeries are invasive and are associated with the risk of mortality and nutritional deficiencies^4^. Furthermore, not all patients sustain the weight reduction^7^. In order to develop safer and more effective therapeutic approaches for the treatment of these diseases, it is important to better understand the molecular mechanisms by which bariatric surgeries exert these beneficial metabolic effects.

There is increasing evidence that metabolic diseases are associated with epigenetic dysregulation. We have previously demonstrated that diet-induced obesity leads to chromatin modifications in the liver of mice^8^ and these can persist even upon the removal of an obesogenic diet^9^. Alteration of promoter methylation of metabolic genes has been observed in muscle tissue from obese patients, and these changes can be reversed after surgery-induced weight loss^10^. Analysis using liver biopsies of NAFLD and normal individuals revealed NAFLD-specific DNA methylation and transcriptional changes for genes involved in metabolism and insulin signaling. These NAFLD-specific DNA methylation signatures were shown to be partially reversible after bariatric surgery^11^.

To investigate the molecular mechanisms underlying the beneficial metabolic effects induced by VSG, we performed genome-wide profiling of chromatin accessibility and gene expression in C57BL/6J wild-type male mice after VSG surgery and compared these profiles to mice with a sham surgery. We demonstrate that diet-induced changes in gene expression and chromatin accessibility can be reversed by VSG.

## Results

### Reversible chromatin accessibility changes after VSG

**Figure 1:**
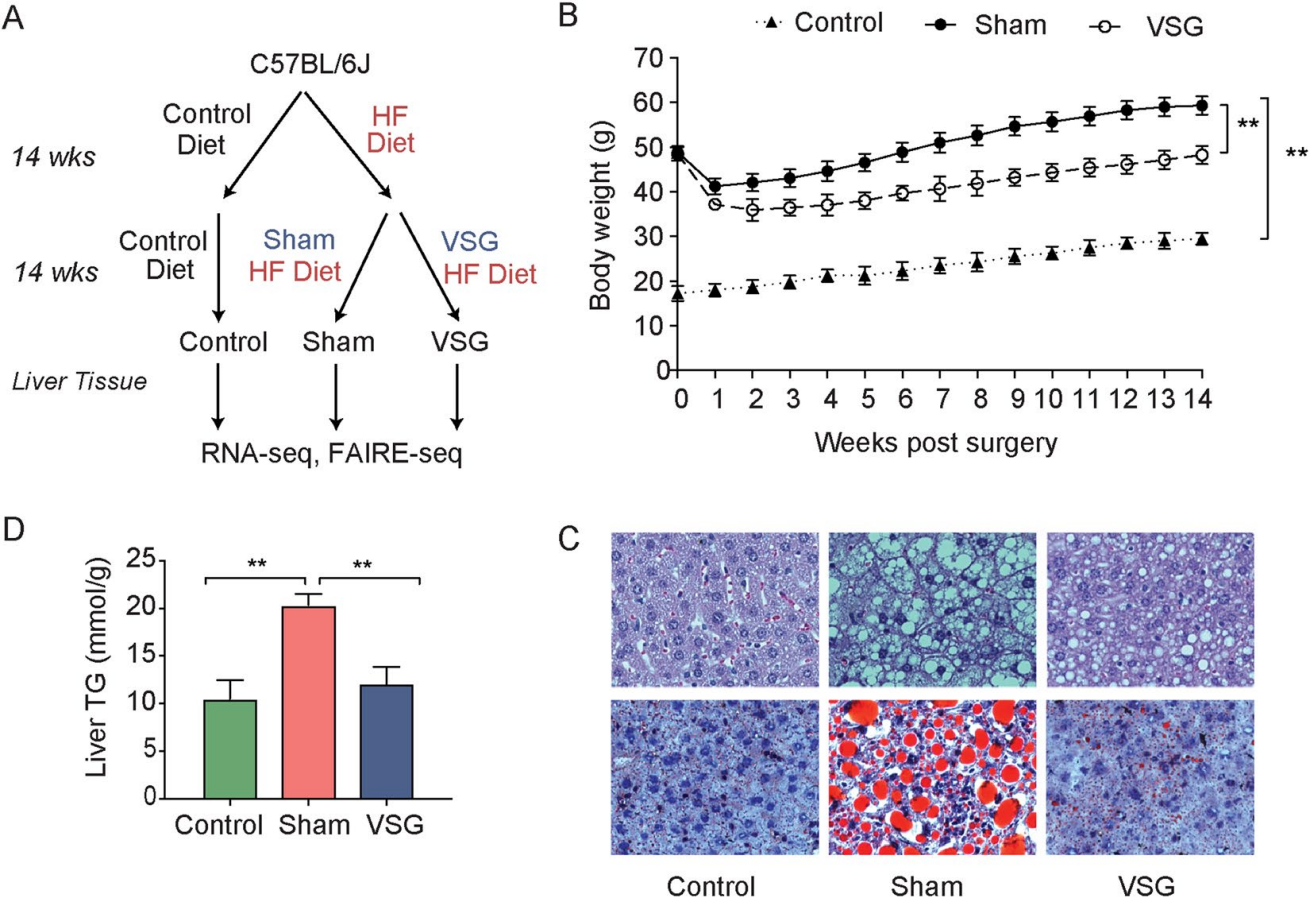
Characteristics of C57BL/6J mice after vertical sleeve gastrectomy (VSG). (**A**) Scheme for mice feeding and surgery for Control, Sham, and VSG group. (**B**) Body weight (grams) over the course of the 14 weeks for Control, Sham, and VSG group. Error bars represent standard error of the mean from 9 to 12 mice in each group. (**Indicates *p* < 0.01) Sham and VSG weights are from Ding, L. *et al*.^12^. (**C**) Hematoxylin and eosin (top) and Oil Red O (bottom) staining from livers of Control, Sham and VSG mice. (**D**) Liver triglyceride (milligrams/g) levels of Control, Sham, and VSG mice. Values are mean ± S.D. (n = 5 per group, **Indicates *p* < 0.01).

We resected liver tissue from wild-type male C57BL/6J mice that underwent Sham or VSG surgery (Fig. 1A). As a control, we used livers from age-matched C57BL/6J mice fed on a control standard chow without surgery (Fig. 1A). As described previously, VSG mice had significant weight loss compared to Sham mice over the course of the study^12^, although VSG mice still weighed more than age-matched control-fed mice (Fig. 1B). Mice that underwent VSG showed reversible changes of liver lipid storage (Fig. 1C) and triglyceride (TG) levels (Fig. 1D). H&E staining showed more lipid droplets in the liver of Sham mice as compared to Control, and the number of lipid droplets decreased in the VSG mice compared to Sham mice (Fig. 1C). Similarly, the hepatic TG levels in Sham mice significantly increased when compared to Control mice (Fig. 1D). In VSG mice, although the hepatic TG levels were higher than Control, they were significantly decreased compared to Sham mice (Fig. 1D). Though the overall weight of VSG mice was greater than Control mice, lipid accumulation and triglyceride levels in liver were reversed by VSG. We next sought to determine the molecular changes associated with these phenotypic changes.

**Figure 2:**
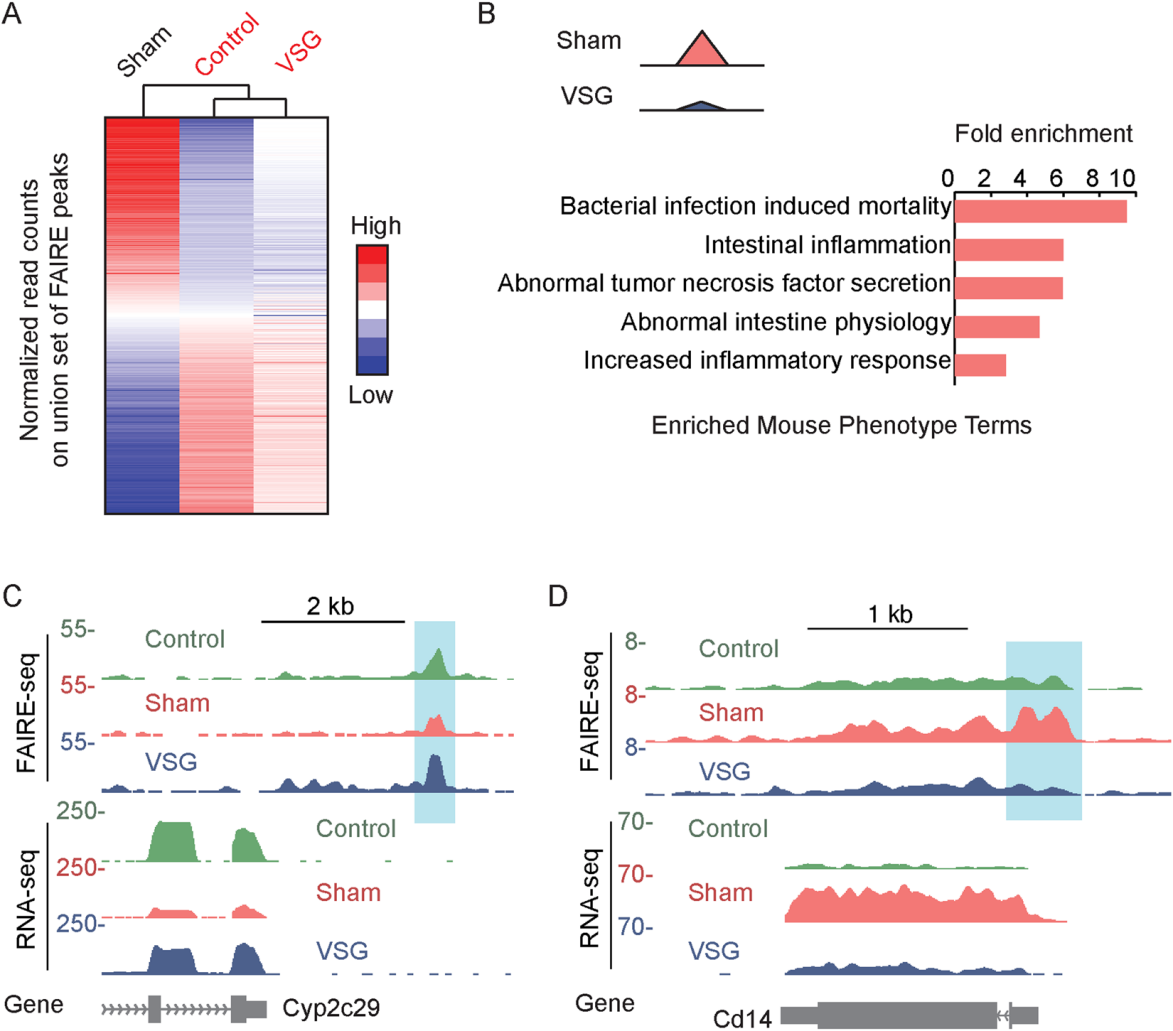
Reversible chromatin accessibility changes after VSG. (**A**) Heatmap of relative read counts at each peak of chromatin accessibility, sorted by relative fold change between Sham and Control group. The dendrogram above the heatmap shows the distance between Control, Sham, and VSG group characterized by read counts at each site. (**B**) Enriched mouse phenotype terms for chromatin sites that are less accessible in VSG mice compared to Sham. (**C** and **D**) Examples of reversible genes and accessible chromatin sites at the *Cyp*2c29 (**C**) and *Cd*14 (**D**) loci.

To understand the regulatory changes induced by VSG, we profiled genome-wide chromatin accessibility in livers of mice that underwent VSG or Sham procedures, as well as Control mice (Fig. 1A). To evaluate the genome-wide signatures of chromatin accessibility in response to VSG, we performed hierarchical clustering on the union set of accessible chromatin sites across the three conditions (Fig. 2A). Overall, the clustering analysis indicated that the chromatin accessibility profiles of mice that underwent VSG were more similar to Control mice than the mice that underwent Sham procedures (Fig. 2A), indicating that chromatin modifications associated with diet-induced obesity are largely reversed by VSG.

In order to understand the molecular networks involved in the VSG response, we began by focusing on regions of the genome that displayed variable accessibility between livers of VSG and Sham mice. Based on GREAT analysis^13^, sites with reduced chromatin accessibility after VSG were enriched for inflammatory phenotypes (Fig. 2B), as well as lipid metabolic diseases (Supplementary Fig. 1). Sites with increased chromatin accessibility after VSG were not enriched for any terms by GREAT analysis. Examples of sites of variable chromatin accessibility and nearby genes are shown in Fig. 2C and D.

### Inflammatory and lipid metabolic genes are altered in mouse liver after VSG

**Figure 3:**
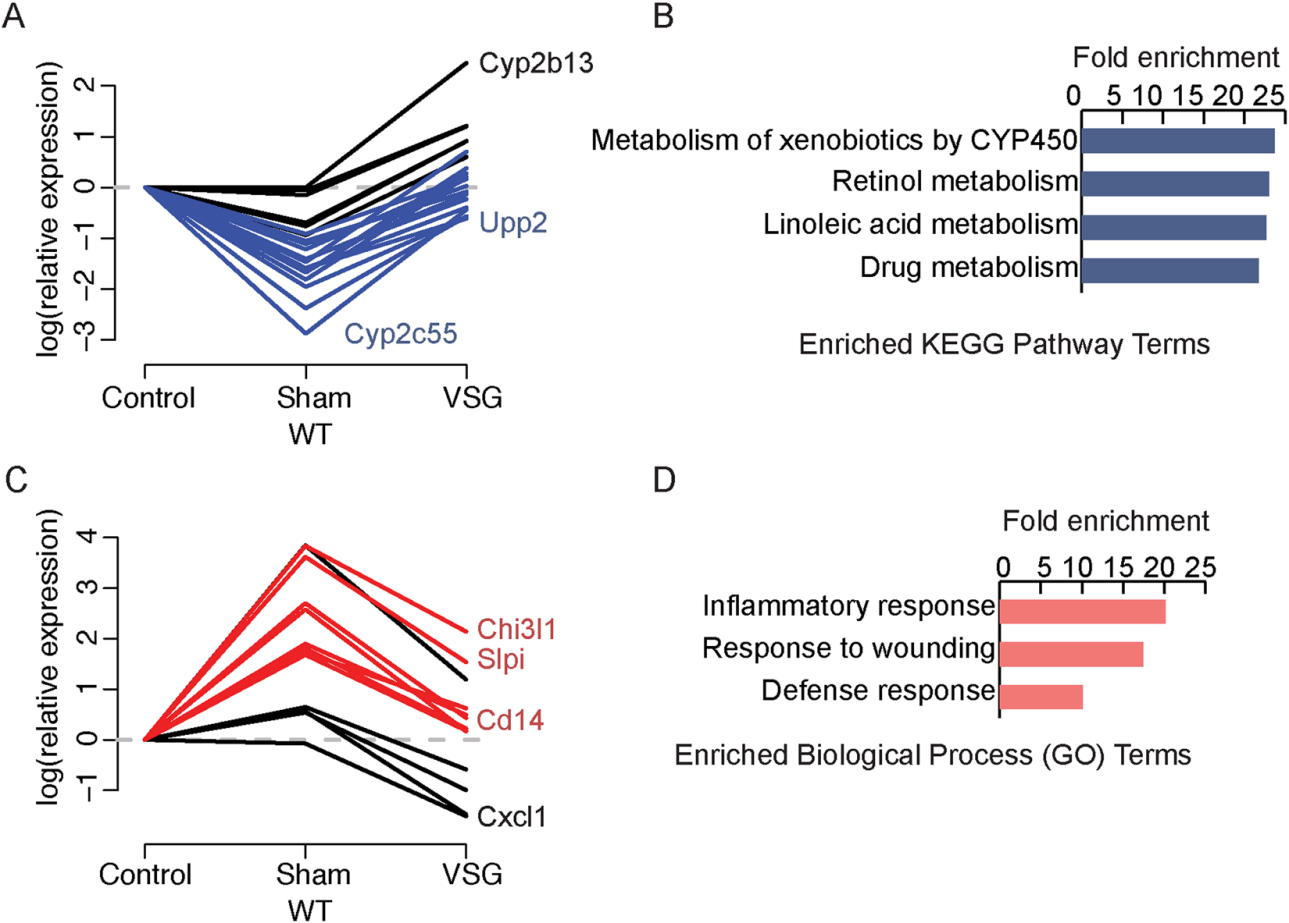
Inflammatory and lipid metabolic genes are altered in mouse liver after VSG. (**A**) Line plot of relative gene expression for genes up-regulated in VSG compared to Sham mice. (**B**) Enriched KEGG Pathway terms for genes up-regulated in VSG compared to Sham mice. (**C**) Line plot of relative gene expression for genes down-regulated in VSG compared to Sham mice. (**D**) Enriched Gene Ontology Biological Process terms for genes down-regulated in VSG compared to Sham mice.

After focusing on putative regulatory regions involved in the VSG response, we next wanted to examine the transcriptional response to VSG. We profiled gene expression by RNA sequencing (RNA-seq) using livers from Control, Sham and VSG mice (Fig. 1A). As with the FAIRE-seq analysis, we focused on genes that were differentially expressed between the VSG and Sham mice. We identified 34 differentially expressed genes between Sham and VSG mice (FDR 10%; Supplementary Table 1). Up-regulated genes in VSG compared to Sham were enriched for metabolic pathways, including retinol and linoleic acid metabolism (Fig. 3A and B). In contrast, genes down-regulated in VSG compared to Sham were enriched for inflammatory and defense response (Fig. 3C and D). This result is consistent with chromatin accessibility changes between VSG and Sham mice, for which sites with reduced accessibility were enriched for inflammatory pathways (Fig. 2B). Using a less stringent threshold to identify differentially expressed genes, we found 355 differentially expressed genes between Sham and VSG mice (*p*-value < 0.05; Supplementary Table 2). With this threshold, up-regulated genes in VSG were still enriched for fatty acid metabolism pathways, and down-regulated genes were still enriched for immune-related pathways (Supplementary Fig. 2), consistent with results obtained using the more stringent threshold (Fig. 3B and D).

When we considered the expression levels of genes of Control mice, 68% (23/34) of the genes differentially expressed between Control and Sham showed “reversible” gene expression trends (blue lines in Fig. 3A and red lines in Fig. 3C). Specifically, several cytochrome P450 (CYP450) genes were down-regulated in Sham compared to Control mice, and reversed in VSG mice (Fig. 3A). Similarly, several immune-related genes, such as *Cd14* (Fig. 2D) and secretory leukocyte peptidase inhibitor (*Slpi*), were up-regulated in Sham compared to Control mice, and reversed in VSG mice (Fig. 3C). We also identified differentially expressed genes between Control and Sham mice (FDR 10% DESeq2; Supplementary Table 3). The down-regulated genes in Sham compared to Control mice are enriched for metabolic pathways, including linoleic acid metabolism (Supplementary Fig. 3A), which were up-regulated in VSG compared to Sham (Fig. 3B). The up-regulated genes in Sham compared to Control mice were enriched for inflammatory and immune response (Supplementary Fig. 3B), which were down-regulated in VSG compared to Sham mice (Fig. 3D). This correspondence of pathways further indicates that VSG can reverse diet-induced transcriptome changes.

### Omega-6 metabolism altered in liver after VSG

**Figure 4:**
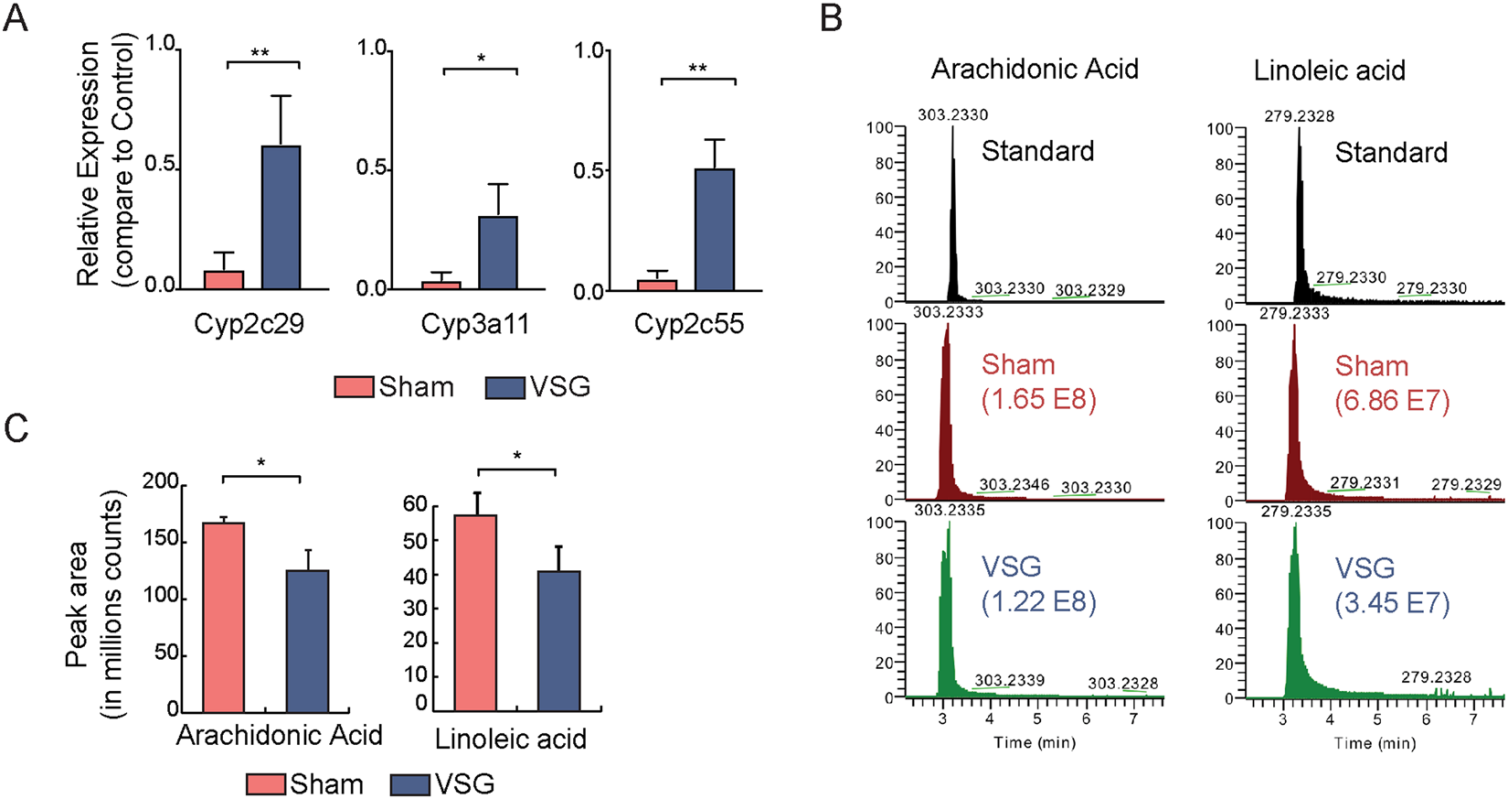
Altered omega-6 metabolism by VSG. (**A**) Relative expression levels of *Cyp*2b29 *Cyp*3a11, and *Cyp*2c55 in Sham and VSG compared to Control mice liver. Expression levels were defined by RT-qPCR. (*Indicates *p* < 0.05, **Indicates *p* < 0.01) (**B**) Representative chromatogram of arachidonic acid and linoleic acid from standards and liver tissues of Sham and VSG mice. Numbers in parentheses represent peak area. (**C**) Peak area from mass spectrometry of arachidonic acid and linoleic acid in Sham and VSG mice liver (*Indicates *p* < 0.05, **Indicates *p* < 0.01; t test).

Several VSG-induced differentially expressed genes were from the CYP450 superfamily. The CYP450 superfamily of proteins act as mono-oxygenases for the synthesis and degradation of endogenous compounds such as fatty acids^14^. Several *Cyp* genes, including *Cyp2c29*, *Cyp2c50*, *Cyp2c55*, *Cyp3a25*, and *Cyp3a59* (Supplementary Fig. 4A), are down-regulated in Sham compared to Control and reversed in VSG. We also noticed the high variability of the FPKM values of different *Cyp* genes, which is consistent with a previous report of diverse expression levels of *Cyp* gene in liver^15^. We further validated the VSG-induced expression changes of three *Cyp* genes with RT-qPCR (Fig. 4A).

These *Cyp* genes we identified as altered in VSG are involved in the metabolism of linoleic acid (LA) and arachidonic acid (AA), two polyunsaturated omega-6 fatty acids. Given the expression changes of lipid metabolic enzymes, we further examined the changes in lipid profiles from Sham and VSG mice by mass spectrometry. We observed reduced levels of TGs in VSG compared to Sham mice (Supplementary Fig. 5A), which is consistent with the measurement by the TG kit (Fig. 1D). We also observed reduced levels of diglyceride (DG) in VSG compared to Sham mice (Supplementary Fig. 5A). The level of total free fatty acids (FFA) in liver was the same between Sham and VSG mice (Supplementary Fig. 5B). However, the levels of both LA and AA were reduced in livers of VSG compared to Sham mice (Fig. 4B and C), which is consistent with the result of increased expression of LA and AA metabolic genes in VSG compared to Sham mice (Fig. 4A). These results indicate that VSG induces changes of omega-6 fatty acid metabolism, although the lipid composition in the diet was the same between Sham and VSG mice^12^.

Another *Cyp* gene altered by VSG is *Cyp7a1*, a key rate-limiting enzyme for bile acid synthesis from cholesterol^16^. *Cyp7a1* is up-regulated in VSG compared to both Sham and Control mice (Supplementary Fig. 4B). Previous studies have demonstrated increased bile acid in both mouse and human after VSG^5, 17, 18^. The increased levels of *Cyp7a1* expression may be an explanation of the bile acid change after VSG.

### Regulatory factors altered by VSG

**Figure 5:**
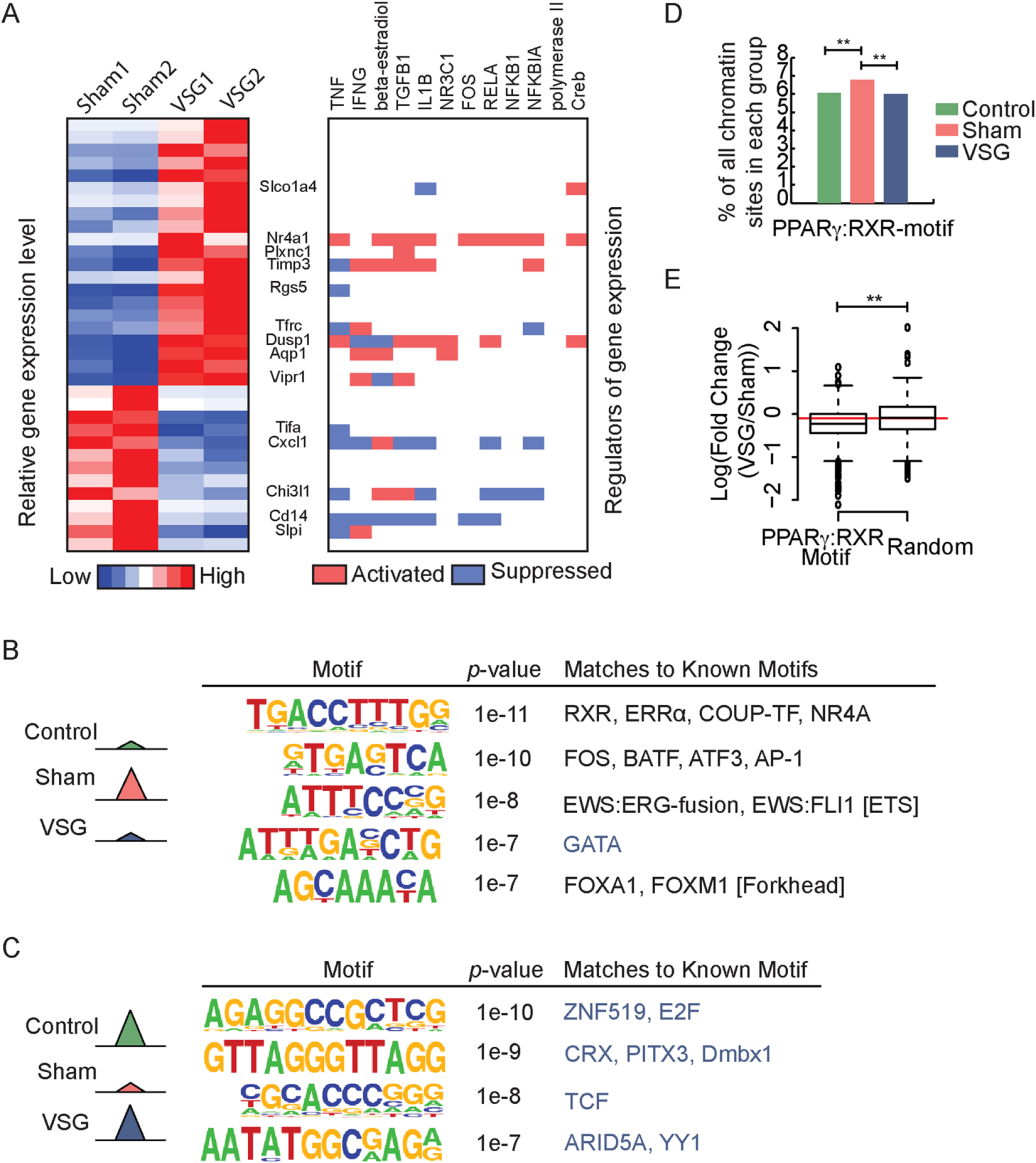
Upstream regulators altered by VSG. (**A**) Heatmap of differentially express genes between Sham and VSG (left), and upstream regulators from Ingenuity Pathways Analysis (right). (**B** and **C**) Identified motifs from reversible chromatin sites that are more (**B**) or less (**C**) accessible in Sham compared to Control and VSG. Numbers in parentheses represent *p*-values of enrichment of motif occurrence in the given set of sequences compared to background, and the percentage of sequences with the motif. (**D**) Percentage of accessible chromatin site in each group that contained PPARγ:RXR motif (**Indicates *p* < 0.01, Fisher’s exact test). (**E**) Fold change (VSG vs Sham) of FAIRE read counts within chromatin sites contain PPARγ:RXR motifs or random chromatin sites (**Indicates *p* < 0.01, Wilcoxon’s rank-sum test).

To identify potential upstream regulators for the differentially expressed genes, we first used Ingenuity Pathway Analysis (IPA) and identified several nuclear receptors and inflammatory factors involved with the gene expression changes after VSG (Fig. 5A). The most enriched upstream regulators were proinflammatory cytokines, TNF-α and IFN-γ^19^. These factors correspond to the VSG-induced regulatory changes and differentially expressed genes in inflammatory response pathways (Figs 2B and 3D).

To identify specific TFs potentially contributing to gene expression changes, we performed *de novo* motif analysis on the sets of chromatin sites with reversible accessibility changes in VSG mice (Fig. 5B,C). The top enriched motif for sites more accessible in Sham and reversed in VSG matched with several nuclear receptors, including retinoid X receptor (RXR) (Fig. 5B). RXR forms a heterodimer with liver X receptors (LXRs), and can act as either a repressor or activator depending on the availability of ligands^20^. LXR-RXR plays a crucial role in lipid homeostasis, and has been shown to regulate *Cyp7a1* ^20, 21^. RXR can also heterodimerize with PPARγ, a key regulator of lipid metabolism (Supplementary Fig. 6A)^22^. To further investigate this, we scanned the whole genome for PPARγ:RXR motif sites and found that the accessible chromatin sites in Sham mice contained significantly more PPARγ:RXR motif sites compared to Control and VSG mice (Fig. 5D). Examining the fold-change of FAIRE-seq read counts further validated the reduction of chromatin accessibility at PPARγ:RXR motif in VSG compared to Sham mice (Fig. 5E). We also checked the PPARγ expression from the different groups. Both isoforms of PPARγ were up-regulated in Sham compared to Control mice, and VSG mice maintained the up-regulated expression of PPARγ (Supplementary Fig. 6B). The induction of PPARγ expression in Sham mice was consistent with previous reports of PPARγ upregulation in fatty liver^23, 24^. However, the consistent expression level of PPARγ in VSG mice does not explain the reduced accessibility at the PPARγ:RXR motif. One possible explanation for the VSG-induced chromatin variation at PPARγ:RXR binding sites could be availability of ligands. Fatty acids, such as linoleic acid, can activate PPAR^25^, which may explain the altered accessibility of PPARγ:RXR binding sites after VSG.

We also searched for enriched motifs at sites less accessible in Sham mice that were reversed in VSG mice. We noticed that many of these sequences were GC rich. Indeed, 59% (103/175) of these sites are at CpG islands. Consistent with this, the *de novo* identified motifs also showed high GC content (Fig. 5C), and matched known motifs have lower matching scores, indicating less confident motif matches (Fig. 5B,C: matched motifs that scored less than 0.7 are colored in blue, while the ones with a score higher than 0.8 are in black).

## Discussion

This study has uncovered molecular pathways and regulatory factors altered in mouse liver after VSG surgery. Our results indicate that inflammatory genes and corresponding regulatory factors are suppressed after VSG, while cytochrome P450-mediated fatty acid metabolic pathways are upregulated after VSG. We further demonstrated that diet-induced changes of gene expression and chromatin accessibility can be reversed by VSG, consistent with the improvement of metabolic phenotypes associated with NAFLD.

The overall body weight for mice that underwent VSG was significantly lower than that of Sham mice, consistent with previous studies on the effectiveness of VSG in persistent weight loss^4, 12^. However, mice that underwent VSG still weighed more than age-matched Control mice 14 weeks post surgery. It may be that a longer period of tracking would show a more significant body weight change. However, within the current experimental design, VSG mice showed reversible hepatic lipid accumulation and triglyceride levels, which further indicates that the beneficial effects of VSG can be independent of overall weight^5^.

Strikingly, one of the most variable pathways altered by VSG involved the *Cyp* genes, which play an important role in lipid metabolism in liver. The upregulation of these genes after VSG was further validated by the reduced levels of linoleic acid in livers of VSG compared to Sham mice. Interestingly, previous studies have demonstrated that dietary conjugated linoleic acid reduces weight gain and fat mass in rodents, although there have been conflicting reports on its effects in humans^26^. Recent studies also show that excessive linoleic acid intake may contribute to chronic inflammation in obese patients^27^. The Sham and VSG mice we used here were fed with the same diet and food intake was similar between the two groups for a majority of the time after surgery^12^. Our results therefore, indicates an alteration of metabolic regulations without the differences of overall change of dietary linoleic acid. In addition, previous studies have shown that polyunsaturated fatty acids, including linoleic acid and arachidonic acid, play important roles in energy balance^28, 29^, although the exact molecular mechanisms remain unclear. This is consistent with the increased energy expenditure in VSG mice we observed previously^12^.

In the post-operative period following VSG, several studies have observed changes in bile acid (BA) composition^30–32^. Besides their functions in lipid absorption and digestion in the intestine, BAs can also act as hormones, interacting with the G-protein-coupled BA receptor 1 (GPBAR-1, also known as TGR5) and the nuclear farnesoid X receptor (FXR)^33^. A recent study demonstrated that the beneficial effects of VSG disappear in FXR knockout mice^17^. We previously demonstrated that TGR5 is necessary for VSG-induced weight loss, insulin sensitizationing, and improvements of fatty liver^12^. A separate study recently demonstrated that TGR5 involved in VSG-induced improvement of glucose homeostasis^34^. We observed the elevated expression of *Cyp7a1*, which encodes a bile acid synthesis enzyme, in livers of mice that underwent VSG compared to Sham and Control. The increased levels of *Cyp7a1* expression may explain the changes in BA after VSG, although a previous study, with different time frames, did not observe the same trend^5^.

In summary, we have shown that VSG modulates a specific group of genes, and corresponding regulatory elements to exert its beneficial metabolic effects. This study may provide novel insight into the future development of effective therapies for obesity, T2D, and NAFLD.

## Research Design And Methods

### Ethics Statement

All the procedures within this study were performed following guidelines from National Institutes of Health. All animal study were approved by the City of Hope Institutional Animal Care and Use Committee (IACUC#14031). All methods were carried out in accordance with the relevant guidelines and regulations.

### Animal and diets

Male C57BL/6J mice were obtained from the Jackson Laboratory and maintained on a standard chow diet ad libitum and a standard 12 h:12 h light/dark cycle until 8 weeks of age. Mice at this age were then given a high-fat diet (Research Diets D12492, 60 kcal% saturated fat) for 14 weeks. These mice were then randomly divided into 2 groups, followed by VSG or Sham surgery. These mice were maintained on the same high-fat diet for 14 weeks after surgery except the recovery during the immediate postoperative period. The mice in the Control group were maintained on standard chow for 28 weeks. During the post-surgery period, the body weights of all mice and food intake were measured weekly. At the end of the experiment, all mice were sacrificed and livers were harvested. Oil Red O and Hematoxylin and eosin (H&E) staining was performed on liver sections by the Pathology Core at the City of Hope using standard procedures.

Triglycerides were assayed by GPO-PAP method (Nanjing Jiancheng Bioengineering Institute). The color intensity of the red dye product is measured photometrically at 510 nm, and is directly proportional to the triglyceride concentration (mmol/L). After adjustment with the protein concentration (gprot/L) in each sample, the reported units of measurement of liver triglycerides are in mmol/g. The Bio-Rad protein concentration assay kit was used for protein measurement.

### VSG surgery

After anaesthetization with isoflurane, the lateral ~80% of the stomach was resected, as previous described^17^. For the Sham surgery group, the stomach was similarly isolated and then subjected to pressure. After the surgery, mice were maintained on a liquid (Osmolite One Cal) diet during the 4-day recovery period. All animals were transitioned onto regular pellets of the designated diet after the recovery period.

### FAIRE-seq

Formaldehyde Isolation of Regulatory Elements (FAIRE) was performed as described previously^8^. FAIRE DNA from two mice of each condition was barcoded and sequenced on the HiSeq 2500 to generate 100 × 100-bp paired-end reads. Sequenced reads were aligned to the mouse mm9 reference genome using bowtie1 and only reads that could be mapped to a unique genomic location were retained^35^. Mapped reads were filtered to exclude improperly paired reads and PCR duplicates. Wiggle tracks were generated for visualization on the UCSC genome browser^36^.

To identify accessible chromatin “peaks” from FAIRE-seq reads, F-seq was used with default parameters and a 250 bp feature length^37^. Irreproducible discovery rate (IDR) analysis was then used to find reproducible peaks between two replicates^38^. A union set of peaks among different conditions was generated by the mergeBed function of BEDTools^39^. To assess global chromatin accessibility across different conditions, read counts for all peaks were normalized^40^, and the conditions were hierarchically clustered with Cluster 3.0^41^ and viewed with TreeView^42^. To identify peaks with variable accessibility between different conditions, we utilized DESeq2 (*p*-value < 0.01)^43^.

### RNA-seq

RNA was extracted from the same livers (two replicates) used for FAIRE using TRIzol (Invitrogen). Ribosomal RNA was depleted from total extracted RNA (Epicenter Ribo-Zero^TM^ magnetic kit, MRZH11124). Eluted RNA was sequenced with Illumina protocols on a HiSeq 2500 to generate 50-bp reads. Sequenced reads were aligned to the mouse mm9 reference genome with TopHat2^44^. Transcript expression was quantified by HTSeq^45^, and DESeq2 was utilized to identify differentially expressed genes. The identification of differentially expressed genes by DEseq2 included adjustment for inter-replicate variations. Genes that differentially expressed in Control vs Sham and Sham vs VSG groups in common were defined as reversible genes. Transcript abundance was quantified as fragments per kilobase of transcript per million fragments mapped (FPKM) using Cufflinks^46^.

### Quantitative real-time PCR

Quantitative Real-Time PCR (qRT-PCR) were performed to validate the expression change of selected genes using additional four liver samples of each condition. Total RNA was extracted from frozen liver tissue using Tri Reagent (Molecular Research Center, Inc), followed by cDNA synthesis using SuperScript First-Strand Synthesis System (Bioland Scientific LLC). qRT-PCR was then performed using SYBR Green qPCR Supermix (Applied Biosystems). The mRNA expression for each gene was normalized to M36B4^17^. Results are shown as relative mRNA level according to 2ˆ(–delta delta CT)^47^. Primer sequences are available in Supplementary Table 4.

### Molecular pathway and motif analysis

Enriched gene ontology terms and KEGG pathways for differentially expressed genes were identified using DAVID (v6.7)^48^. Bonferroni-corrected *p*-values < 0.1 were considered statistically significant. Gene interaction networks were generated by submitting differentially expressed genes to the Ingenuity Pathways Analysis (IPA) (www.ingenuity.com)

To characterize the enriched biological functions of sites of accessible chromatin, we used genomic coordinates of accessible chromatin sites as input for Genomic Regions Enrichment of Annotations Tool (GREAT)^13^. Region-gene associations were defined using default parameters and significant associations for “Mouse Phenotype” and “Disease Ontology” were included. Only terms with a false discovery rate (FDR) < 0.05 were reported.

*De novo* motif discovery was carried out using HOMER (version 4.8) with default parameters^49^. Motifs were matched to known motifs with higher scores being better matches. STORM^50^ was further used to scan for occurrences of the PPARγ:RXR motif genome wide. Putative binding sites were defined by motif occurrences within accessible chromatin regions identified in C57BL/6J mouse liver, similar to what has been reported before^51^.

### Lipid extraction and Mass spectrometry

Livers from additional three mice of each condition was used for lipid extraction followed by mass spectrometry. Mouse liver tissue was flash frozen in liquid nitrogen immediately after sacrifice, then a portion of 100 mg was transferred to tubes containing glass beads and homogenized at 4 °C for 6 min in 0.25 mL PBS containing 2 mM EDTA and 100 μM t-butylated hydroxytoluene^52^. The homogenate was extracted using 0.75 mL of chloroform/methanol (2:1), concentrated in a nitrogen evaporator then dissolved in a volume of buffer B (10% acetonitrile in isopropanol containing 10 mM ammonium formate), proportional with the initial tissue weight for analysis.

The lipids (10 µL injection) were separated with a Dionex Ultimate 3000 UHPLC on a Phenomenex Kinetex 2.1 × 100 mm C18 column with 1.7 µm particle size with a gradient of isopropanol/acetonitrile/water over 25 min from 60% buffer A (40% acetonitrile in 10 mM ammonium formate in water) to 100% buffer B over 15 min then 4 min at 100% B^53^. Lipids eluting from the UHPLC column were measured with a Thermo Orbitrap Fusion mass spectrometer operated in DDA in either positive or negative ion mode, scanning from 210–1410 at 120000 resolution with a 1 sec duty cycle during which the maximum number of precursor ions were selected by the quadrupole, with an isolation width of 1.4 Da, for HCD fragmentation and their product ion spectra subsequently measured in the Orbitrap at 30000 resolution. The data was then analyzed by *in silico* product ion database search using Thermo Lipid search 4.1.

### Statistical analysis

Data in this study is presented as mean ± standard deviation or standard error of the mean. Statistical tests were performed using Student’s t test or one way ANOVA, and *p* < 0.05 was considered statistical significant.

## Electronic supplementary material

Supplementary Documents
