# Supplementary material for: Vertical sleeve gastrectomy reverses diet-induced gene-regulatory changes impacting lipid metabolism
Source: Sci Rep. 2017 Jul 13;7:5274. doi: 10.1038/s41598-017-05349-2 (PMC5509746; doi:10.1038/s41598-017-05349-2)
Supplement: Supplementary file 1 — Supplementary Documents [file 41598_2017_5349_MOESM1_ESM.pdf]

Vertical sleeve gastrectomy reverses diet-induced gene-regulatory changes impacting lipid metabolism

Juan Du[1,2], Jingyan Tian[1,3], Lili Ding[1], Candi Trac[1], Brian Xia[1], Siming Sun[1], Dustin E. Schones[1,2], Wendong Huang[1,2]

[1]Department of Diabetes Complications and Metabolism, Beckman Research Institute, City of Hope, Duarte, CA, USA.

[2]Irell & Manella Graduate School of Biological Sciences, City of Hope, Duarte, CA, USA.

[3]Shanghai Clinical Center for Endocrine and Metabolic Diseases, Shanghai Institute of Endocrine and Metabolic Diseases, Department of Endocrinology and Metabolism, China National Research Center for Metabolic Diseases, Ruijin Hospital, Shanghai Jiao Tong University School of Medicine, Shanghai, China.

Juan Du and Jingyan Tian contributed equally to this work. Correspondence and requests for materials should be addressed to W.H. (WHuang@coh.org) or D. E. S. (email: dschones@coh.org) or J. T. (tianjypaper@163.com)

Supplementary Figure 1

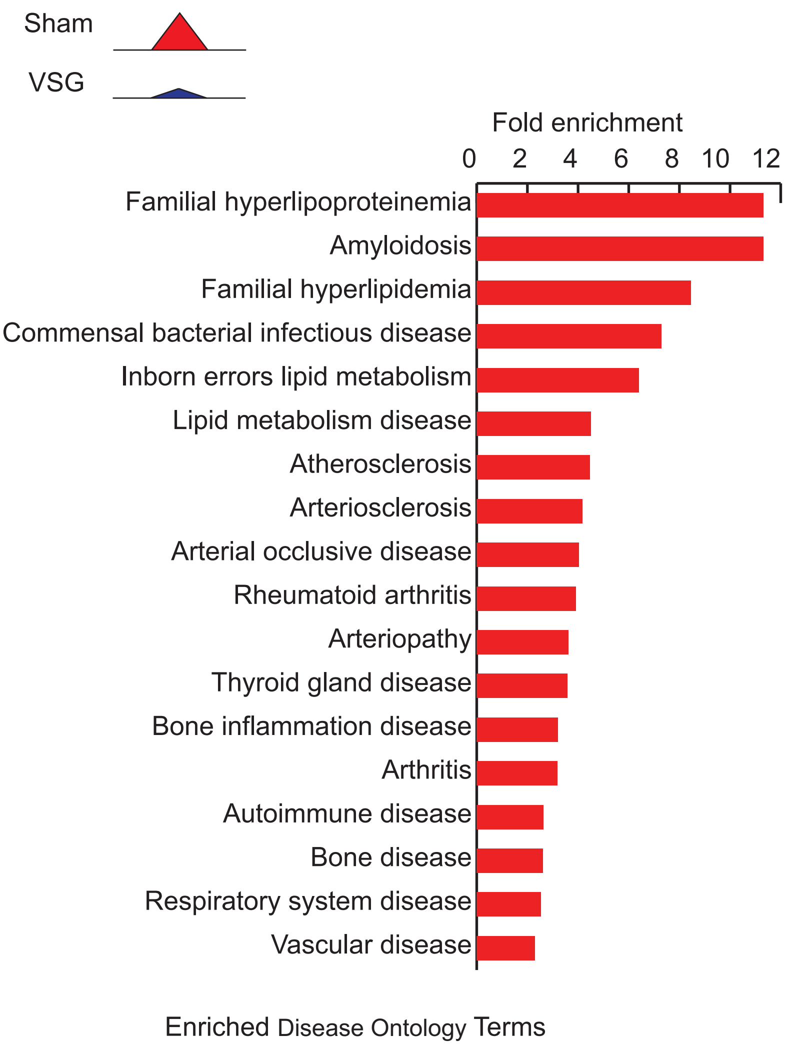

Supplementary Figure 1 - Function of chromatin sites that are less accessible in VSG compared to sham. Functional analysis was done using GREAT.

Supplementary Figure 2

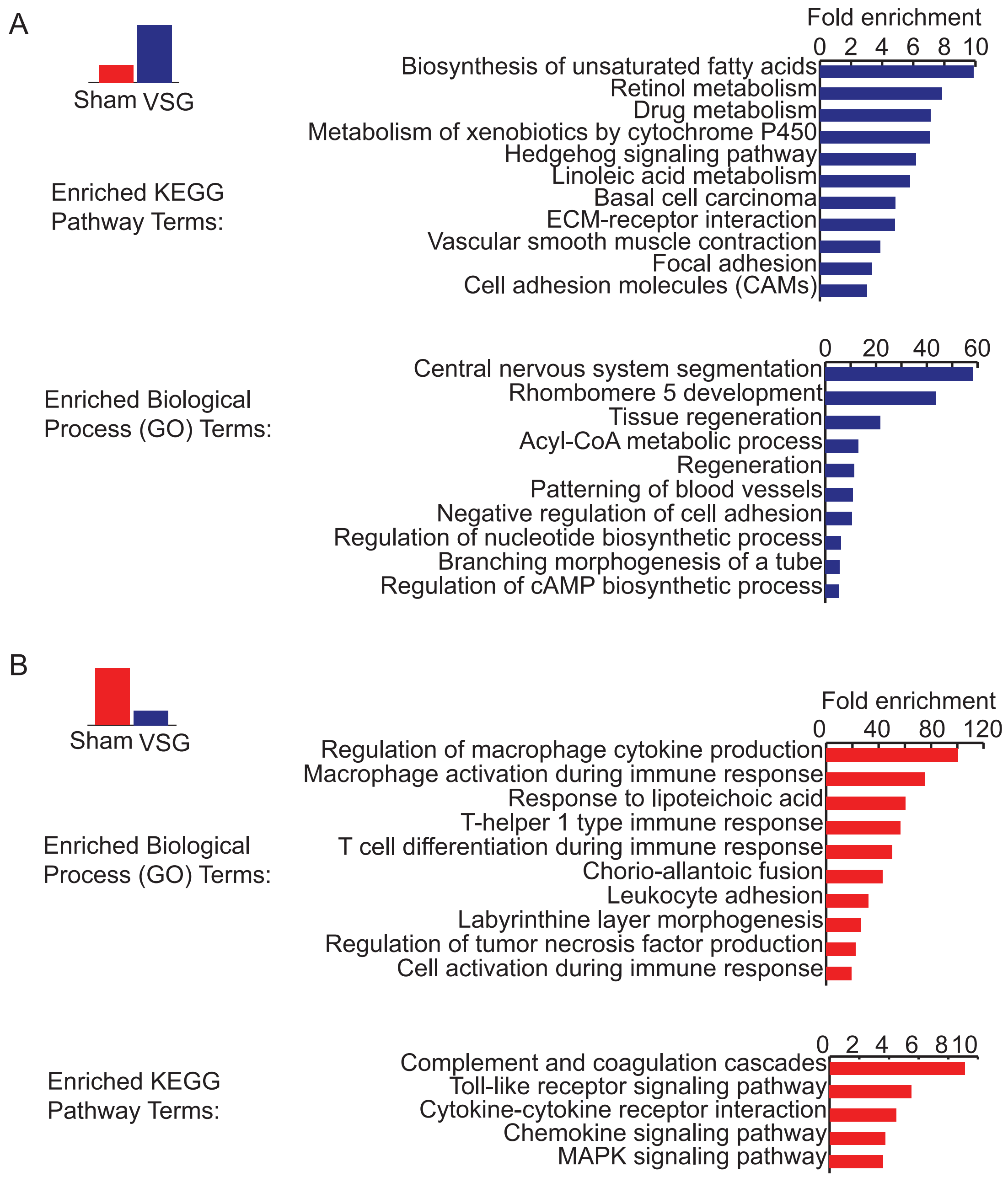

Supplementary Figure 2 - Function of differential expressed genes (DESeq2, p-value < 0.05) in VSG mice compared to sham mice. (A-B) Enriched KEGG pathways and biological process for genes up-(A) or down-(B) regulated in VSG mice compared to sham. Pathway analysis was done using DAVID, and Bonferroni-corrected *p* values smaller than 0.05 were considered as statistically significant association.

Supplementary Figure 3

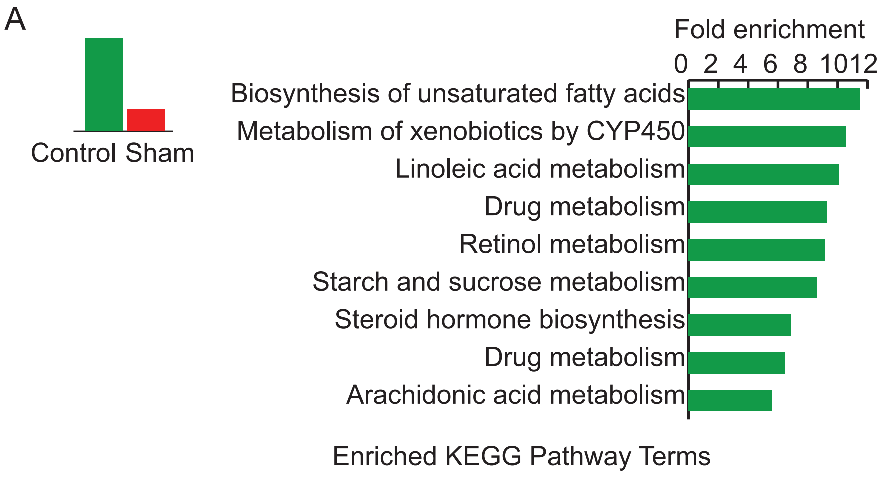

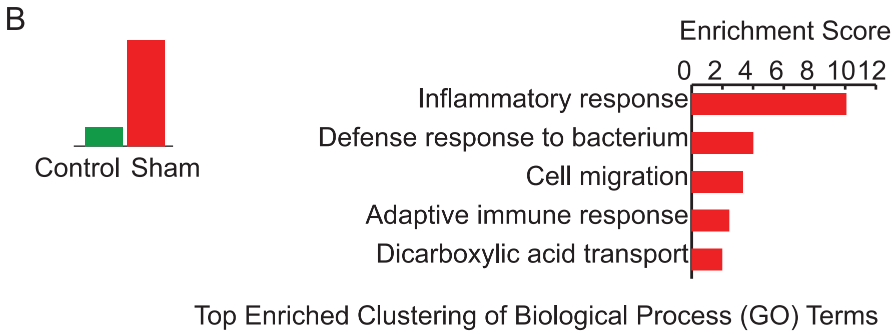

Supplementary Figure 3 - Function of differential expressed genes in sham mice compared to control mice. (A) Enriched KEGG pathways for genes down-regulated in sham mice. (B) Enriched biological process for genes up-regulated in sham mice. Pathway analysis was done using DAVID.

Supplementary Figure 4

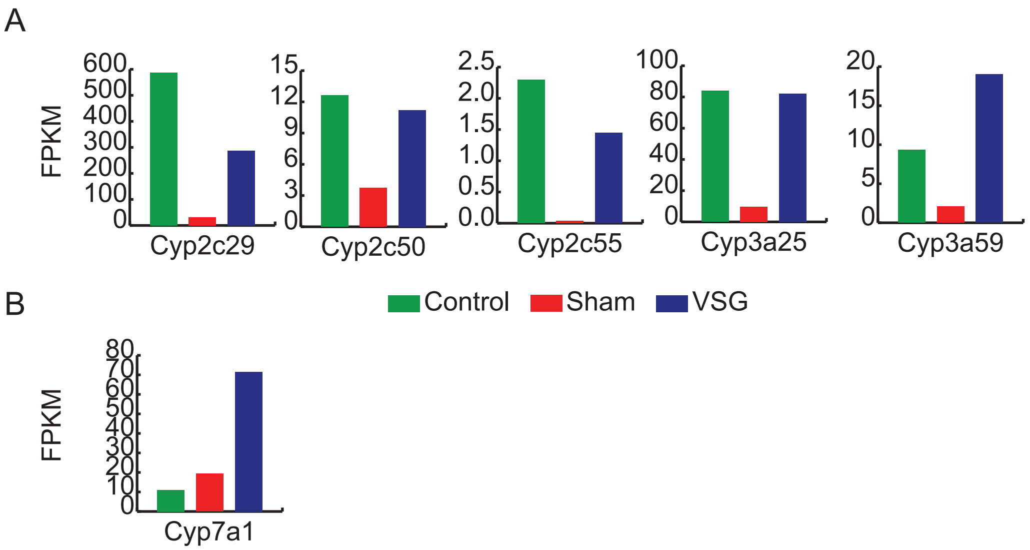

Supplementary Figure 4 - Expression of Cyp genes altered after VSG. Expression level of Cyp genes in the pathways of linoleic acid metabolism (A) and bile acid synthesis (B). FPKM: fragments per kilobase of exon per million fragments mapped.

Supplementary Figure 5

A

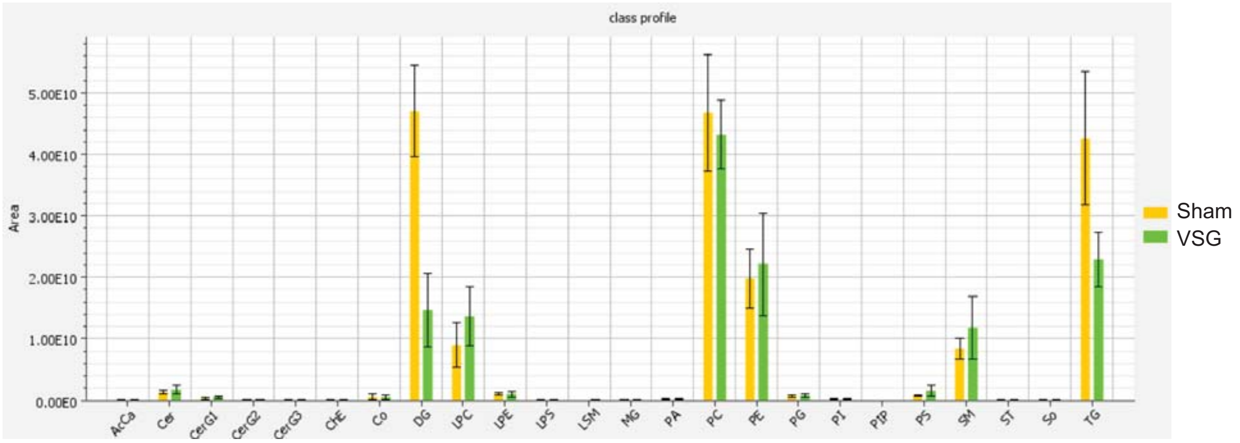

B

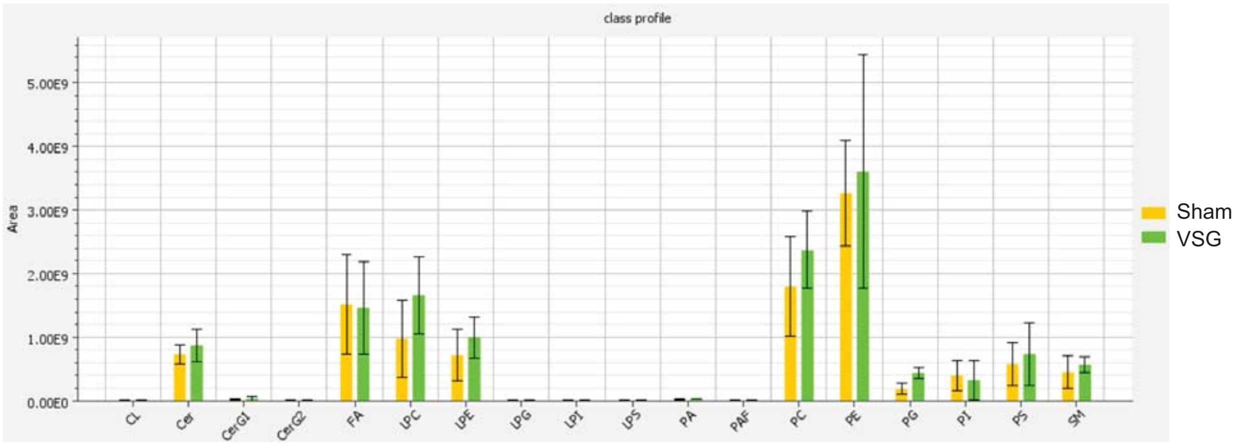

Supplementary Figure 5 - Lipidomics class profile. Lipid class profile in positive (A) or negative (B) ion mode.

Supplementary Figure 6

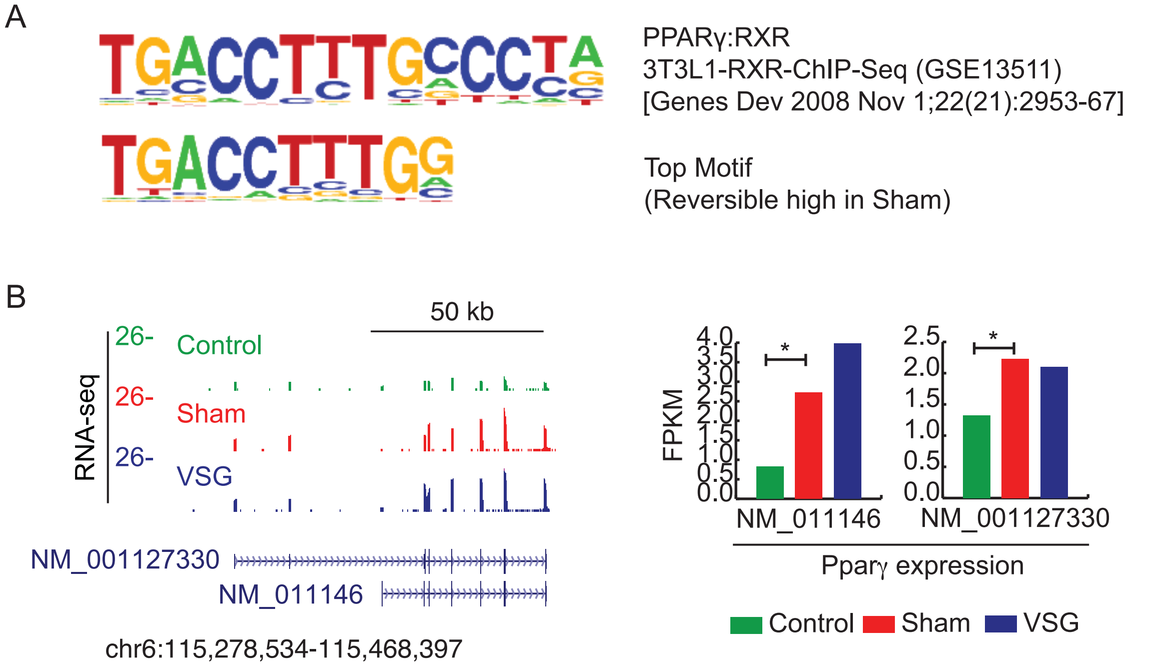

Supplementary Figure 6 - PPARγ in VSG. (A) Top motif in reversible chromatin sites matched with PPARγ:RXR binding site. Top: known PPARγ:RXR binding motif. Bottom: top enriched motif for reversible chromatin sites more accessible in sham liver. (B) Expression level of PPARγ in three conditions. Left: UCSC genome browser tracks of RNA-seq data at PPARγ locus. Right: FPKM for PPARγ isoforms.

Supplementary Table 1: Differential expressed genes between Sham and VSG in wild type mice (DESeq2, FDR 10%)

Supplementary Table 1a: Genes down-regulated in VSG compared to Sham

Gene Name	*Gene ID*	*CTL_FPKM*	*SHAM_FPKM*	*VSG_FPKM*
Ypel4	NM_001005342	0	4.58097	0.229726
Gm7694	NM_001198955	6.69646	11.5488	2.41653
Chi3l1	NM_007695	0.206519	14.1172	2.50874
Cxcl1	NM_008176	120.004	111.54	26.4341
Cd14	NM_009841	2.22812	34.6514	3.48942
Efna3	NM_010108	0.0221391	0.714621	0.0999818
Slpi	NM_011414	0.302078	14.8894	1.77135
Orm3	NM_013623	12.4856	70.3935	23.4248
Isyna1	NM_023627	9.52138	58.4269	11.9168
Alpk1	NM_027808	0.965085	5.62657	1.18058
Slc10a6	NM_029415	0.397244	0.7735	0.0136674
Tifa	NM_145133	24.3755	46.7212	13.5325
Rtn4rl2	NM_199223	0.161963	3.3582	0.210218

Supplementary Table 1b: Genes up-regulated in VSG compared to Sham

Gene Name	*Gene ID*	*CTL_FPKM*	*SHAM_FPKM*	*VSG_FPKM*
Cyp3a59	NM_001105160	9.32061	2.07647	18.9959
Apold1	NM_001109914	0.196258	0.038223	0.640645
Upp2	NM_001289660	7.39208	0.595162	4.16106
Aqp1	NM_007472	15.7325	3.13974	10.5957
Cyp2b13	NM_007813	0	0	1.06301
Fcna	NM_007995	11.2468	5.58481	28.1297
Rgs5	NM_009063	2.59942	2.22418	8.89018
Nr4a1	NM_010444	2.8016	1.03863	5.19713
Lipg	NM_010720	7.62194	1.47292	4.09023
Timp3	NM_011595	1.25802	0.0923087	0.661725
Tfrc	NM_011638	3.54274	3.33896	12.1613
Vipr1	NM_011703	3.2989	1.0881	3.03071
Dusp1	NM_013642	27.8672	8.17442	32.9919
Plxnc1	NM_018797	1.42445	0.274261	1.11328
Cyp2c55	NM_028089	2.29858	0.035197	1.45295
Slco1a4	NM_030687	10.8128	1.68243	13.2938
Clec2h	NM_053165	1.14262	0.133785	1.54728
Acot3	NM_134246	15.8951	2.92186	23.2769
Colec10	NM_173422	1.75799	0.645813	2.14698
Col14a1	NM_181277	3.2388	0.997466	3.3461
1500017E21Rik	NR_033510	15.1399	3.59155	13.2343

Supplementary Table 2: Differential expressed genes between Sham and VSG in wild type mice (DESeq2, p-value < 0.05)

Supplementary Table 2a: Genes down-regulated in VSG compared to Sham (132 genes)

Gene Name	*Gene ID*	*CTL_FPKM*	*SHAM_FPKM*	*VSG_FPKM*
Slc13a5	NM_001004148	0.934472	3.5427	0.988564
Tacc2	NM_001004468	2.88311	2.08079	0.483146
Ypel4	NM_001005342	0	4.58097	0.229726
E2f8	NM_001013368	11.3782	8.86621	4.03892
Rasl10b	NM_001013386	0.0371898	0.880947	0.282369
Qsox1	NM_001024945	36.0353	71.0204	33.9136
Nlrp12	NM_001033431	30.7357	26.3536	15.6334
Zfhx2	NM_001039198	1.49092	2.13203	1.15041
Cerkl	NM_001048176	3.73011	5.93283	2.00681
Nos1ap	NM_001109985	1.35309	1.90692	0.474979
Atg16l2	NM_001111111	8.17458	5.89162	2.76497
Lepr	NM_001122899	1.75259	0.940422	0.267917
Gm7694	NM_001198955	6.69646	11.5488	2.41653
Serpina3i	NM_001199940	0.19964	0.566767	0.0447235
E030018B13Rik	NM_001256311	9.12985	2.27634	0.287673
Cebpd	NM_007679	12.2246	6.24996	3.21215
Chi3l1	NM_007695	0.206519	14.1172	2.50874
Socs3	NM_007707	21.7684	29.6399	9.15643
Cp	NM_007752	286.515	996.386	476.059
Cyb561	NM_007805	8.15066	19.78	7.9394
Egfr	NM_007912	45.4451	69.6033	35.5749
Cxcl1	NM_008176	120.004	111.54	26.4341
Foxq1	NM_008239	17.385	8.11613	3.9906
Id3	NM_008321	55.7682	36.3802	17.9278
Il17ra	NM_008359	9.50567	10.8882	5.55909
Irg1	NM_008392	0.0777055	0.486266	0.115204
Lcn2	NM_008491	228.722	4890.34	1328.69
Mmp8	NM_008611	0.07429	0.358168	0.0628887
Mt2	NM_008630	5209.66	3330.08	386.779
Ngp	NM_008694	0.0236198	1.06042	0.180801
Serpine1	NM_008871	1.27917	8.48876	2.74686
Cxcl2	NM_009140	0.416498	1.62258	0.390407
Cd14	NM_009841	2.22812	34.6514	3.48942
Ccr1	NM_009912	0.0944706	0.658071	0.127656
Cx3cr1	NM_009987	0.120876	1.29638	0.461365
Cyp21a1	NM_009995	11.6484	19.3747	6.86763
Efna3	NM_010108	0.0221391	0.714621	0.0999818
Fga	NM_010196	943.351	1505.82	608.748
Hmox1	NM_010442	37.1049	31.4926	15.5659
Icam1	NM_010493	7.29406	18.1452	8.09727
Ier5	NM_010500	1.25434	4.19217	2.08195
Il18bp	NM_010531	3.41349	44.4527	15.9346

Itga4	NM_010576	1.6105	2.52216	1.19475
Ly6d	NM_010742	1.42716	139.846	51.5176
Orm2	NM_011016	66.9744	5666.11	1070.2
Saa3	NM_011315	18.5867	3327.81	250.861
Apcs	NM_011318	106.036	3349	921.325
C4a	NM_011413	27.4167	33.5746	11.7736
Slpi	NM_011414	0.302078	14.8894	1.77135
Sox9	NM_011448	1.94905	3.88728	1.65634
Gadd45g	NM_011817	199.358	39.4876	16.5248
Tlr2	NM_011905	0.664174	3.35208	1.17307
Map3k1	NM_011945	2.49925	3.12586	1.60976
Mapk13	NM_011950	0.183044	0.95215	0.227843
Adrb3	NM_013462	2.87095	7.81329	4.42218
Cpe	NM_013494	0.0572429	1.09347	0.19837
Hspb1	NM_013560	51.983	74.3729	35.0402
Orm3	NM_013623	12.4856	70.3935	23.4248
Pnp	NM_013632	27.1789	30.9655	15.9599
Pla2g7	NM_013737	1.10264	3.60945	1.63211
Pdk4	NM_013743	19.2609	9.77763	2.62169
Map3k6	NM_016693	2.59479	1.07286	0.265574
Neu3	NM_016720	0.830108	0.9113	0.374654
S100a11	NM_016740	15.3021	116.32	55.4284
Nfil3	NM_017373	45.0352	20.8076	11.7577
Cxcl13	NM_018866	1.97595	2.22045	0.622171
Dynll1	NM_019682	5.32353	16.8856	8.14422
Ikbke	NM_019777	3.46955	12.5988	6.43951
Litaf	NM_019980	77.8569	115.074	53.0509
B3galt1	NM_020283	29.8254	22.9806	11.5095
Smpd3	NM_021491	0.555027	4.62028	1.83697
Sdf2l1	NM_022324	20.8961	41.6576	16.983
Kng1	NM_023125	613.962	1262.92	628.553
Sult1e1	NM_023135	0.653887	3.39464	0.427103
Ubd	NM_023137	0.357363	24.0856	8.92518
Isyna1	NM_023627	9.52138	58.4269	11.9168
Cpne8	NM_025815	3.28182	6.06762	2.75712
Zc3h13	NM_026083	6.76436	6.64533	3.62295
Wfdc2	NM_026323	7.39025	70.4009	33.6909
Tmem86a	NM_026436	2.54265	16.6218	8.6166
9530077C05Rik	NM_026739	0.191747	0.942817	0.119448
Chac1	NM_026929	10.6408	12.7546	3.83427
2010003K11Rik	NM_027237	50.083	58.1949	30.7854
Alpk1	NM_027808	0.965085	5.62657	1.18058
Osgin1	NM_027950	118.995	192.799	101.496
Tmco6	NM_028036	4.1529	7.6494	3.59315
Ddx28	NM_028038	13.6718	28.1356	10.7347
Bmper	NM_028472	0.20928	1.18895	0.1904
Irak3	NM_028679	0.812769	2.02258	0.708407

Scara5	NM_028903	27.1348	15.8993	4.04156
8430419L09Rik	NM_028982	14.4355	19.7254	9.78655
Ddit4	NM_029083	11.1575	7.42857	2.82035
Manf	NM_029103	23.2013	47.8932	25.2773
Slc10a6	NM_029415	0.397244	0.7735	0.0136674
Ly6k	NM_029627	0	1.62382	0.0922505
Nol3	NM_030152	0.111748	1.9674	0.897126
Nrg4	NM_032002	26.821	18.3115	8.74618
Myo7b	NM_032394	0	0.130176	0.0176522
D17H6S56E-5	NM_033075	0.910895	3.27085	1.19852
Bcl3	NM_033601	42.4523	38.1502	15.9457
Prelp	NM_054077	8.26213	21.9882	6.0384
Steap4	NM_054098	214.963	280.672	67.0959
Atp6v0a4	NM_080467	0.0475408	0.312474	0.00453601
Mvp	NM_080638	17.0722	58.1922	30.8833
Caskin2	NM_080643	3.401	7.27831	3.93647
Prss8	NM_133351	3.4095	4.83169	2.3422
Ier3	NM_133662	1.44168	12.4666	2.48811
Fbxo31	NM_133765	47.3708	18.5812	10.675
Clp1	NM_133840	16.5133	15.3713	8.40881
Syt12	NM_134164	1.73169	6.44573	2.60633
Tnip2	NM_139064	5.15367	5.09368	2.74982
Lgi4	NM_144556	0.0473251	0.272808	0.029857
Serpina10	NM_144834	278.096	667.902	234.154
Tifa	NM_145133	24.3755	46.7212	13.5325
Fam129b	NM_146119	1.04015	6.02839	3.1313
Atoh8	NM_153778	8.47638	6.93448	2.34477
Fbf1	NM_172571	22.0108	7.35223	3.73463
Zc3h12d	NM_172785	0.63495	1.65853	0.682636
Fndc3b	NM_173182	5.91593	11.7984	4.45846
A2m	NM_175628	0.0333337	16.0872	0.159154
Slc41a2	NM_177388	14.3317	21.8062	10.7429
AA986860	NM_177604	2.40817	1.41633	0.460879
Sbno2	NM_183426	10.8099	14.737	6.22311
Fam211a	NM_198861	0.537035	3.39232	0.596322
Rtn4rl2	NM_199223	0.161963	3.3582	0.210218
Eif4ebp3	NM_201256	145.725	51.2856	22.4391
Ccdc120	NM_207202	0.19831	2.51846	0.840217
A330023F24Rik	NR_015566	2.30764	1.08908	0.474139
D7Ertd143e	NR_028425	7.44066	3.19166	0.978867
Gm15348	NR_033546	0.196091	0.664149	0.132101
Gm4262	NR_040518	0.23055	0.758076	0.172287
Neat1	NR_131212	0.624551	0.615284	0.153415

Supplementary Table 2b: Genes up-regulated in VSG compared to Sham (223 genes)

Gene Name	*Gene ID*	*CTL_FPKM*	*SHAM_FPKM*	*VSG_FPKM*
Tmem204	NM_001001183	4.12711	0.860403	1.86892
Gpr126	NM_001002268	0.159723	0.0195069	0.138241
Cxcl12	NM_001012477	10.6667	7.23705	14.4062
Capn11	NM_001013767	0.190086	0.0231559	0.594187
Akr1c19	NM_001013785	60.1524	12.9014	48.1477
N4bp2	NM_001024917	2.49186	0.785624	1.64054
Trp53i11	NM_001025246	1.70653	0.677987	1.63341
Gm129	NM_001033302	12.1493	3.94335	8.62955
Fam38b	NM_001039485	0.117064	0.0223809	0.0912968
Gpr116	NM_001081178	2.96694	0.399534	1.13986
Lilra5	NM_001081239	0.400609	0.468306	1.27818
Cdh18	NM_001081299	0.0301841	0.38112	1.13442
Neto2	NM_001081324	0.152523	0.0216326	0.123162
Cyp2a22	NM_001101467	1.62082	3.01388	8.45772
F830016B08Rik	NM_001101475	6.7297	2.64423	6.90451
Cyp3a59	NM_001105160	9.32061	2.07647	18.9959
Apold1	NM_001109914	0.196258	0.038223	0.640645
Lrrc32	NM_001113379	1.85928	1.02914	1.88285
Acss3	NM_001142804	1.56837	1.35245	3.99655
Psma8	NM_001163609	0.454493	0.305816	0.998892
Syde2	NM_001166064	1.87633	0.685311	1.3191
Zbed6	NM_001166552	4.01647	1.3577	3.96922
Cyp2c50	NM_001167877	12.6357	3.72625	11.2085
Dcn	NM_001190451	29.7063	8.25237	34.3044
Macf1	NM_001199136	0.181	0.0357685	0.192618
Lcp1	NM_001247984	10.652	2.2796	8.77728
Prlr	NM_001253781	8.17543	6.0049	17.5054
Lin7a	NM_001284329	0.356748	0.145688	0.836297
Upp2	NM_001289660	7.39208	0.595162	4.16106
Fdx1	NM_001301728	3.26241	2.162	3.96438
Acta2	NM_007392	0.320024	0.086167	0.589014
Aqp1	NM_007472	15.7325	3.13974	10.5957
Bgn	NM_007542	25.5773	12.4288	23.6615
Bmp2	NM_007553	2.92857	0.751931	1.99038
Bmp5	NM_007555	1.71027	0.545839	1.70355
Cyp2b13	NM_007813	0	0	1.06301
Cyp2c29	NM_007815	588.055	31.6565	288.401
Cyp7a1	NM_007824	11.0338	19.4157	71.5578
Dnase1l3	NM_007870	5.71959	1.49681	4.76697
Fcna	NM_007995	11.2468	5.58481	28.1297
Flt4	NM_008029	2.16957	0.672851	1.55013
Fosb	NM_008036	0.0455333	0.048328	4.75592
Frat1	NM_008043	1.85581	1.71926	3.05699
Fzd8	NM_008058	1.86196	1.97455	4.14344
Gas1	NM_008086	2.84312	1.32571	3.8767

Gja4	NM_008120	3.57002	0.655069	1.70485
Grid1	NM_008166	0.134435	0.168007	0.674389
Gsta2	NM_008182	11.4015	0.65549	3.19342
Hcn3	NM_008227	2.74467	1.05499	3.29827
Hpgd	NM_008278	31.081	25.8023	76.0056
Hspg2	NM_008305	0.882853	0.292609	0.640565
Igfbp3	NM_008343	5.95176	1.54106	2.98221
Kcna2	NM_008417	0.0717817	0.213414	0.490957
Kcnb1	NM_008420	0.24734	0.0355987	0.128157
Klf2	NM_008452	1.93276	1.54202	4.35881
Lama1	NM_008480	0.193364	0.0907044	0.38707
Lamb1	NM_008482	0.668794	0.316264	1.01311
Ltb	NM_008518	0.162163	0.2232	1.04812
Mmp12	NM_008605	0.0212273	0.278202	0.886127
Mrc1	NM_008625	2.75768	1.63614	3.70495
Npr1	NM_008727	1.74305	0.702637	1.4508
Ntn1	NM_008744	0.708703	0.258038	1.05487
Ntrk2	NM_008745	0.0727124	0.39192	0.871798
Prkg2	NM_008926	0.0281166	0.0291267	0.163171
Rcn1	NM_009037	1.43972	0.617666	1.31687
Rgs4	NM_009062	0.451915	0.0993994	0.397804
Rgs5	NM_009063	2.59942	2.22418	8.89018
Scd2	NM_009128	0.514188	0.352327	0.746043
Tgfbi	NM_009369	10.371	6.33941	14.0191
Thbd	NM_009378	1.46231	0.218891	0.957805
Wee1	NM_009516	5.94449	2.28492	5.02619
Adra2b	NM_009633	0.375951	0.286611	0.799427
Bcl6	NM_009744	4.80655	11.7021	25.5495
Chic1	NM_009767	0.245871	0.171884	0.399022
Ccnd2	NM_009829	0.383456	0.184679	0.467805
Cdh5	NM_009868	2.04944	0.960924	2.79911
Col3a1	NM_009930	1.52617	1.98517	4.12379
Cd55	NM_010016	2.90087	0.792285	1.99217
S1pr3	NM_010101	0.216092	0.182139	0.524203
Egr2	NM_010118	0.0507222	0.0985353	0.37002
Epor	NM_010149	0.416452	0.103043	0.705353
Erbb4	NM_010154	0.0679041	0.113878	0.337643
Fos	NM_010234	2.08549	1.61868	50.2889
Gbp1	NM_010259	0.138068	0.0880223	1.02615
Gem	NM_010276	0.991039	0.372144	1.24387
Gja1	NM_010288	1.09379	0.389525	1.43134
Ednra	NM_010332	0.608885	0.309603	0.869803
H2-Ob	NM_010389	0.104232	0.113536	0.384168
Nr4a1	NM_010444	2.8016	1.03863	5.19713
Icam2	NM_010494	0.929425	0.591488	1.80553
Cyr61	NM_010516	2.41489	5.02193	15.7442
Jun	NM_010591	20.0023	12.6715	53.0686

Kdr	NM_010612	2.22553	0.411308	1.47809
Klf4	NM_010637	0.213003	0.270742	0.831503
Mafb	NM_010658	36.2641	8.45354	19.9447
Lipg	NM_010720	7.62194	1.47292	4.09023
Neb	NM_010889	0.520314	0.400081	0.912506
Oit3	NM_010959	4.97513	1.19797	3.12594
Abcb1a	NM_011076	0.39618	0.533753	1.82265
Prlr	NM_011169	1.05886	1.71346	4.96297
Rgs16	NM_011267	36.8463	23.8803	74.9613
Sod3	NM_011435	13.1622	8.01749	15.4147
Sox7	NM_011446	0.394971	0.0914362	0.373663
Tie1	NM_011587	2.42769	0.66648	1.48598
Timp3	NM_011595	1.25802	0.0923087	0.661725
Tnc	NM_011607	0.0425422	0.00796124	0.173458
Tfrc	NM_011638	3.54274	3.33896	12.1613
Vipr1	NM_011703	3.2989	1.0881	3.03071
Vwf	NM_011708	0.326795	0.125525	0.409964
Adam23	NM_011780	1.56573	0.429374	1.24455
Car14	NM_011797	20.8404	11.301	19.134
Papss2	NM_011864	29.3881	10.3031	34.0064
Spry4	NM_011898	1.78732	0.868573	2.85165
Angptl2	NM_011923	2.44603	0.67966	2.50887
Acot1	NM_012006	88.5006	11.8079	26.0392
Pam	NM_013626	2.57261	1.23851	2.9818
Dusp1	NM_013642	27.8672	8.17442	32.9919
Sema5b	NM_013661	0.328601	1.35061	3.19763
Podxl	NM_013723	0.767106	0.118935	0.460417
Cldn5	NM_013805	1.57494	0.91123	2.39387
Cyp2g1	NM_013809	0.257474	0.0762001	0.456918
Heyl	NM_013905	0.229258	0.0501734	0.226284
Col5a1	NM_015734	0.519051	0.275972	0.648142
Rgs1	NM_015811	0.834051	0.720028	3.17576
Nes	NM_016701	0.0990786	0.0388822	0.161019
Clec4f	NM_016751	9.39609	10.8242	33.4954
Ppp1r3c	NM_016854	3.84553	7.37176	14.317
Ramp1	NM_016894	1.37412	0.438321	1.23701
Tlr5	NM_016928	3.66708	3.21713	5.89532
Calcrl	NM_018782	1.15518	0.394263	1.41247
Plxnc1	NM_018797	1.42445	0.274261	1.11328
Ramp2	NM_019444	6.44742	2.11966	5.76709
Gdf2	NM_019506	4.25817	1.56883	2.95537
Ccl24	NM_019577	0.270413	0.209721	1.20341
Tspan7	NM_019634	4.7068	2.31176	5.53042
Slc22a4	NM_019687	1.21206	0.579125	1.23502
Foxo3	NM_019740	4.14119	2.00664	3.75137
Cyp3a25	NM_019792	83.9468	9.81594	82.2288
Hhip	NM_020259	0.150117	0.0262242	0.218393

Arhgap31	NM_020260	0.622771	0.144055	0.371871
Ehd3	NM_020578	8.34343	2.82495	5.43602
Shank3	NM_021423	0.782153	0.218324	0.503928
Kcnk5	NM_021542	8.92771	4.88106	9.19895
Fads3	NM_021890	1.37678	1.0712	1.90377
Ddr2	NM_022563	0.164635	0.176633	0.359994
Wfdc1	NM_023395	1.57166	0.360644	1.94953
Lrat	NM_023624	1.5921	0.862803	2.01412
Wnt2	NM_023653	0.805657	0.16295	0.770382
Jam2	NM_023844	1.85239	0.404418	1.2597
Arl4d	NM_025404	15.3342	10.7179	21.9783
Slc16a9	NM_025807	0.544094	0.179874	0.562344
Clec14a	NM_025809	1.10969	0.331997	1.06121
Gatm	NM_025961	1.14057	1.44975	2.76738
Mxra7	NM_026280	0.875593	0.239035	1.00507
Plxnd1	NM_026376	1.65873	0.772444	1.65036
Rcn3	NM_026555	2.25854	1.58999	3.22
Gpihbp1	NM_026730	14.0372	3.63214	9.88167
Myct1	NM_026793	0.682412	0.248205	0.849083
Sult1c2	NM_026935	1.59669	2.42143	6.64132
Esam	NM_027102	5.07272	1.52091	3.26009
Cyp2c55	NM_028089	2.29858	0.035197	1.45295
Rspo3	NM_028351	1.00462	0.538203	1.44459
Dock8	NM_028785	2.34915	1.95878	4.24982
Clec4g	NM_029465	27.36	8.9884	20.0081
Ppp1r3g	NM_029628	12.5304	0.553316	1.44847
Ptprb	NM_029928	2.62646	0.46736	1.37047
Chst15	NM_029935	1.78116	0.387083	1.01399
Cygb	NM_030206	2.90657	1.57007	3.91669
Slco1a4	NM_030687	10.8128	1.68243	13.2938
Plvap	NM_032398	12.725	6.93409	16.9618
Clec2h	NM_053165	1.14262	0.133785	1.54728
Cadm3	NM_053199	0.477003	0.230259	0.62407
Adcy4	NM_080435	0.804812	0.256627	1.05351
Eltd1	NM_133222	1.24704	0.314518	1.02875
N4bp2l1	NM_133898	28.073	13.1717	25.7316
Gstt3	NM_133994	24.8857	7.22079	17.4666
Acot3	NM_134246	15.8951	2.92186	23.2769
Acot4	NM_134247	15.3397	7.82957	18.3317
Slc22a27	NM_134256	0.160019	1.12629	2.53157
Stab2	NM_138673	2.26374	1.93138	3.98763
Sdpr	NM_138741	2.40937	0.558086	1.44848
Emr4	NM_139138	0.225293	0.524523	1.32462
Spsb4	NM_145134	0.565558	0.375521	1.49717
Angptl6	NM_145154	8.52519	13.9033	13.3039
Cd300ld	NM_145437	0.541854	0.979646	2.37063
Setd4	NM_145482	2.54755	2.73696	5.75263

Tox	NM_145711	2.56261	1.76557	3.09556
Slc22a26	NM_146232	0.278216	0.21215	0.890669
Trhde	NM_146241	0.304302	0.0497978	0.251234
Lrit1	NM_146245	2.5147	1.48348	2.73696
Hpse	NM_152803	1.04109	0.493526	1.20642
Plk2	NM_152804	2.58547	2.05993	3.73179
Mmrn2	NM_153127	1.14249	0.366148	0.823641
Abca8a	NM_153145	11.8746	6.77412	16.365
Gimap6	NM_153175	3.70983	1.18946	3.1912
Ppat	NM_172146	5.80236	3.33003	5.33918
She	NM_172530	0.395893	0.0741399	0.242726
Lhfpl2	NM_172589	0.702604	0.291734	0.599684
Slc22a29	NM_172776	0.0495365	0.332993	1.21536
Btnl9	NM_172793	0.307811	0.0602166	0.450384
Colec10	NM_173422	1.75799	0.645813	2.14698
Mamdc2	NM_174857	0.413331	0.201939	0.680416
Impg2	NM_174876	0.318163	0.489075	1.03392
Mmd2	NM_175217	0.297584	2.48272	6.65961
Exph5	NM_176846	1.64718	1.18206	1.87611
Lrtm1	NM_176920	0.0710776	0.179497	0.443534
Slc17a4	NM_177016	7.23817	7.84627	15.9747
Arhgef15	NM_177566	1.49123	0.376653	1.03662
Slc26a10	NM_177615	1.07918	0.475	1.76516
Fam167a	NM_177628	0.0201181	0.0493303	0.252392
Vsig4	NM_177789	2.77177	5.94643	14.8835
Slc25a48	NM_177809	2.04745	1.46777	3.39986
Lcorl	NM_178142	1.10752	0.403663	0.89205
Timd4	NM_178759	0.820029	1.04217	2.844
Col14a1	NM_181277	3.2388	0.997466	3.3461
Fam167b	NM_182783	4.91739	1.42905	3.56154
Gprin3	NM_183183	1.09384	0.701731	1.56636
Usp2	NM_198091	21.6524	7.46362	12.256
Ugt1a9	NM_201644	23.172	3.0444	8.50645
1700030C10Rik	NR_015521	0.840302	0.21691	0.794023
1500017E21Rik	NR_033510	15.1399	3.59155	13.2343
AW549542	NR_045702	3.74654	3.86873	7.81692
9030619P08Rik	NR_108041	2.18966	5.43825	12.9143

Supplementary Table 3: Differential expressed genes between sham and control mice (DESeq2, FDR 10%)

Supplementary Table 3a: Genes up-regulated in sham compared to control (308 genes)

Gene Name	*Gene ID*	*CTL_FPKM*	*SHAM_FPKM*	*VSG_FPKM*
BC048546	NM_001001179	3.60042	17.9088	10.1145
AI413582	NM_001002895	2.91967	10.9979	8.80643
Slc13a5	NM_001004148	0.934472	3.5427	0.988564
Arhgap30	NM_001005508	0.275024	0.833719	1.1011
Sept11	NM_001009818	0.91324	2.95191	1.93671
Rasl10b	NM_001013386	0.0371898	0.880947	0.282369
Cenpj	NM_001014996	0.496864	1.30313	0.969402
Shc2	NM_001024539	0.0985988	0.925251	0.215979
Pls1	NM_001033210	0.0206803	0.520487	0.216077
Uap1l1	NM_001033293	2.65641	18.6915	15.4478
Dnahc1	NM_001033668	0.00213384	0.0644253	0.024172
Pnldc1	NM_001034866	0.658948	4.14653	6.50203
Csf1r	NM_001037859	2.27885	6.21642	9.65982
Ccdc17	NM_001037916	0.672533	2.3471	2.21003
Arrdc3	NM_001042591	6.01176	30.2862	55.7596
Gm6484	NM_001080940	14.2995	43.0166	71.4906
Ermp1	NM_001081213	5.99521	12.4549	9.94167
Mn1	NM_001081235	1.44296	3.09218	3.03107
Cdh18	NM_001081299	0.0301841	0.38112	1.13442
Cul9	NM_001081335	0.597218	1.39273	1.40825
Wdr67	NM_001081396	1.04157	3.24042	2.33851
Zfp655	NM_001083958	3.32162	11.2545	9.29011
Myom3	NM_001085509	0.0646513	0.693829	0.191341
Fam131c	NM_001085513	2.20572	7.89779	6.07846
2510049J12Rik	NM_001101431	4.3338	9.46062	7.88077
H2-Q4	NM_001143689	12.3916	29.619	24.19
Srcrb4d	NM_001160366	0.331502	1.6092	1.11686
Eme2	NM_001163102	2.58609	6.85289	6.12837
Pcsk5	NM_001190483	1.04783	2.00562	1.72862
Bin2	NM_001270537	0.338254	1.34191	1.64134
Ces2h	NM_001272045	0	0.555963	0.58504
Ddx60	NM_001293783	0.301717	0.82193	0.885786
Ms4a4a	NM_001310331	0.0341355	1.11306	0.264529
Ntpcr	NM_001310688	0.541237	2.1256	1.66728
Afp	NM_007423	0.383839	1.44269	1.08487
Btc	NM_007568	0.238171	1.01521	1.03884
C1qa	NM_007572	13.8224	35.8973	45.6859
C1qc	NM_007574	10.9792	33.921	43.8764
Anxa2	NM_007585	4.15127	25.9163	25.2614
Chi3l1	NM_007695	0.206519	14.1172	2.50874
Cidea	NM_007702	0.0517071	10.9776	16.581
Col8a1	NM_007739	0.0616048	0.520727	0.211589

Cyb561	NM_007805	8.15066	19.78	7.9394
Cybb	NM_007807	0.555274	2.68873	2.8158
Hn1	NM_008258	6.5293	15.0558	12.8596
Ifit2	NM_008332	0.214421	0.816667	0.745407
Il10rb	NM_008349	2.92565	6.44772	5.53583
Itgb2	NM_008404	0.709773	2.02077	3.0969
Lgals1	NM_008495	14.6031	102.138	90.733
Blnk	NM_008528	0.500533	2.29562	1.35759
Man2b2	NM_008550	9.06104	21.6567	19.054
Mas1	NM_008552	0	0.459828	0.268606
Mcm2	NM_008564	0.713794	2.28894	1.9119
Mthfd2	NM_008638	0.609186	2.22577	1.50974
Ngp	NM_008694	0.0236198	1.06042	0.180801
Ntrk2	NM_008745	0.0727124	0.39192	0.871798
Serpine1	NM_008871	1.27917	8.48876	2.74686
Ppt1	NM_008917	9.53063	20.6762	16.6341
Rac2	NM_009008	0.840181	2.37906	2.09984
Rad51b	NM_009014	0.468896	9.16909	10.2582
St8sia3	NM_009182	0.197584	1.06703	0.708412
Ptk6	NM_009184	0.0845049	0.626992	0.401511
Serpinb9	NM_009256	0.876482	2.91822	3.16557
Stk10	NM_009288	0.31237	1.17549	1.01735
Tert	NM_009354	0.59254	1.66762	1.98956
Tnfaip2	NM_009396	2.57606	6.59933	5.73461
Cd5l	NM_009690	7.18881	29.8768	54.8432
C1qb	NM_009777	11.3534	39.1332	45.3554
Cd14	NM_009841	2.22812	34.6514	3.48942
Entpd2	NM_009849	1.23467	3.67776	3.14513
Chi3l3	NM_009892	0.0677255	1.52601	0.971364
Ccr1	NM_009912	0.0944706	0.658071	0.127656
Ccr2	NM_009915	0.114687	1.06095	0.660639
Ccr5	NM_009917	0.263755	1.11075	1.59182
Col15a1	NM_009928	1.293	2.92281	1.91115
Col4a1	NM_009931	3.55064	11.0616	5.41678
Cx3cr1	NM_009987	0.120876	1.29638	0.461365
Efna3	NM_010108	0.0221391	0.714621	0.0999818
Emr1	NM_010130	0.666428	2.59503	4.20971
Samd9l	NM_010156	1.33433	3.8796	3.40259
Fcer1g	NM_010185	6.28583	22.6554	20.0042
Fcgr1	NM_010186	0.26126	1.50285	1.2351
Fcgr3	NM_010188	1.66555	5.40695	4.69531
Fgr	NM_010208	0.356882	1.62313	0.977264
S1pr2	NM_010333	2.25827	5.88508	3.49562
Hexb	NM_010422	5.46798	13.863	12.0077
Icam1	NM_010493	7.29406	18.1452	8.09727
Ier5	NM_010500	1.25434	4.19217	2.08195
Il18bp	NM_010531	3.41349	44.4527	15.9346

Laptm5	NM_010686	2.49181	6.87473	7.80136
Ly6d	NM_010742	1.42716	139.846	51.5176
Ly86	NM_010745	1.52527	6.46742	6.30734
Marco	NM_010766	3.29807	16.6361	12.6525
Mpeg1	NM_010821	5.18529	27.8141	20.7266
Ncf2	NM_010877	0.290691	1.04846	0.985088
Ntn3	NM_010947	0.61616	1.39803	1.38486
Orm2	NM_011016	66.9744	5666.11	1070.2
Reg3b	NM_011036	0.0894474	8.18446	1.13935
Prkca	NM_011101	0.474201	1.30824	1.35152
Pld3	NM_011116	7.79154	16.4873	12.5738
Lgals3bp	NM_011150	26.0035	69.8277	53.4683
Lgmn	NM_011175	16.7483	62.3659	40.9056
Prtn3	NM_011178	1.56169	40.0818	21.5136
Rbl1	NM_011249	0.0847429	0.4404	0.344479
Reg3g	NM_011260	0	2.75448	0.713687
Saa3	NM_011315	18.5867	3327.81	250.861
Apcs	NM_011318	106.036	3349	921.325
Sfpi1	NM_011355	1.0238	3.46479	4.41978
Slc23a1	NM_011397	12.9376	27.8295	28.6004
Slpi	NM_011414	0.302078	14.8894	1.77135
Siglec1	NM_011426	0.216814	0.637507	1.08963
Serpinb8	NM_011459	0.470627	1.74944	2.00224
Sdc3	NM_011520	2.1462	4.68808	9.02202
Tap2	NM_011530	6.85389	22.2268	17.7382
Tnfsf12	NM_011614	6.05196	16.8753	13.726
Tyrobp	NM_011662	4.44158	24.0863	29.9838
Ucp2	NM_011671	11.8208	56.6181	38.8854
Trpv2	NM_011706	0.189658	0.978813	1.02853
Twf2	NM_011876	1.26039	3.46698	3.02948
Tlr2	NM_011905	0.664174	3.35208	1.17307
Usp18	NM_011909	3.47675	9.8766	7.9169
Abcd2	NM_011994	0.739491	3.77116	6.78856
Adrb3	NM_013462	2.87095	7.81329	4.42218
Psmb9	NM_013585	8.39511	55.667	44.4402
Lyz1	NM_013590	1.74397	9.57168	5.18078
Orm3	NM_013623	12.4856	70.3935	23.4248
S100a8	NM_013650	5.88595	27.1465	10.7152
Sema5b	NM_013661	0.328601	1.35061	3.19763
Pla2g7	NM_013737	1.10264	3.60945	1.63211
Gyg	NM_013755	2.38041	5.83068	5.51314
Elane	NM_015779	0.0315714	0.979416	0.450718
Isg15	NM_015783	6.57564	30.9555	18.5089
Cldn1	NM_016674	7.3398	25.525	16.4878
Gpc1	NM_016696	2.62088	15.4256	16.3916
S100a11	NM_016740	15.3021	116.32	55.4284
Cntnap1	NM_016782	0.649353	6.60602	2.82922

Gipc2	NM_016867	0.0681529	1.6068	0.586413
Hmgn2	NM_016957	10.596	31.937	25.7016
Slc7a8	NM_016972	0.230954	0.823715	1.0489
Lyz2	NM_017372	10.0738	55.0578	40.2942
Gipc1	NM_018771	3.1717	8.00346	6.64057
Slc1a4	NM_018861	0.29945	2.91512	2.46122
Pdzrn3	NM_018884	1.39706	3.63901	3.171
Robo1	NM_019413	0.254254	0.877511	0.976084
Aif1	NM_019467	1.43933	8.18666	7.32042
Gas6	NM_019521	13.7111	59.6394	39.8085
Extl1	NM_019578	1.04365	6.3686	6.22986
Dynll1	NM_019682	5.32353	16.8856	8.14422
Nme4	NM_019731	1.00082	5.2059	4.4567
Nupr1	NM_019738	1.93591	9.02574	5.15397
Ikbke	NM_019777	3.46955	12.5988	6.43951
Ube2l6	NM_019949	38.0451	109.133	65.0473
Fgf21	NM_020013	8.26486	83.2606	75.8123
Cxcl10	NM_021274	1.34103	5.71047	4.20854
Zbp1	NM_021394	1.16968	7.22908	4.0982
Smpd3	NM_021491	0.555027	4.62028	1.83697
Tmem8	NM_021793	7.28852	14.5488	12.7062
Tmem176b	NM_023056	3.07708	12.0609	8.0466
Ubd	NM_023137	0.357363	24.0856	8.92518
Isyna1	NM_023627	9.52138	58.4269	11.9168
Uba7	NM_023738	1.64503	4.05263	4.16926
Hdhd3	NM_024257	8.65398	21.0469	25.5287
Fabp4	NM_024406	16.5481	56.7002	40.2949
Wbscr27	NM_024479	3.19324	6.85848	5.62024
Nenf	NM_025424	9.47656	37.2287	25.7104
Tm4sf20	NM_025453	0.0556125	1.05921	0.458309
Smim8	NM_025471	8.51566	20.7736	15.7711
Acot11	NM_025590	1.04472	2.418	2.79254
Ntpcr	NM_025636	2.30722	7.93399	6.93418
Fuca2	NM_025799	1.32015	4.2433	3.07874
Herc6	NM_025992	1.71623	3.41679	3.20716
Wfdc2	NM_026323	7.39025	70.4009	33.6909
Plgrkt	NM_026362	2.2682	8.30853	7.11066
Tmem86a	NM_026436	2.54265	16.6218	8.6166
Ggct	NM_026637	3.37182	9.77877	8.0429
Fam114a1	NM_026667	6.58167	17.0809	13.6444
Golt1a	NM_026680	8.48378	18.8262	15.0214
Mogat1	NM_026713	0.608189	4.26825	5.16311
Zfp579	NM_026741	1.44967	4.22459	2.85838
Gsdmd	NM_026960	13.9504	28.1799	22.8005
Enho	NM_027147	10.0679	33.0741	23.1861
2010107G23Rik	NM_027251	0.535193	2.76361	2.17991
Alpk1	NM_027808	0.965085	5.62657	1.18058

Tha1	NM_027919	2.96873	11.8299	6.92492
Slc35f2	NM_028060	0.351123	3.37441	2.2292
Cyth4	NM_028195	0.70493	2.94774	1.88962
Bmper	NM_028472	0.20928	1.18895	0.1904
Ms4a6c	NM_028595	0.710609	2.56967	1.68569
Arsg	NM_028710	1.50876	7.55061	4.85134
Mterfd3	NM_028832	0.92085	3.79701	3.18332
Hsf2bp	NM_028902	0.152606	1.08173	0.618713
Pck2	NM_028994	1.02264	2.99665	1.55117
Ocstamp	NM_029021	0.440396	2.0644	0.830524
4930444A02Rik	NM_029037	1.26609	3.37882	2.58883
Trim14	NM_029077	4.60022	12.0513	11.4393
Kbtbd11	NM_029116	0.0333283	0.282669	0.555518
1700019G17Rik	NM_029331	1.06913	2.82966	2.33078
Ms4a4c	NM_029499	0.0355937	0.644328	0.270704
Gbp8	NM_029509	0.342356	1.28666	1.19635
Tmem98	NM_029537	4.1796	16.7221	12.6077
Ly6k	NM_029627	0	1.62382	0.0922505
Srxn1	NM_029688	10.6953	22.7121	23.1157
Rab36	NM_029781	0.164355	0.808708	0.759562
2310014L17Rik	NM_029809	0.362709	1.52574	0.440074
Prelid2	NM_029942	0.581337	4.40165	2.78416
Nxpe2	NM_030069	3.54983	16.3834	15.4665
Dhx58	NM_030150	2.16129	6.52325	5.74957
Nol3	NM_030152	0.111748	1.9674	0.897126
Ak7	NM_030187	0.110907	0.831823	0.387109
Abcg3	NM_030239	0.133889	0.823792	1.03522
Dpp7	NM_031843	11.7623	33.1227	22.4762
Myo7b	NM_032394	0	0.130176	0.0176522
D17H6S56E-5	NM_033075	0.910895	3.27085	1.19852
Chrm3	NM_033269	0.385154	1.25211	1.03313
Dqx1	NM_033606	1.60316	4.07317	3.32497
Fam126a	NM_053090	1.08066	2.98824	2.18633
Gpnmb	NM_053110	0.0401983	2.76016	0.69424
Myo1f	NM_053214	0.15289	0.658063	0.86005
Slc13a3	NM_054055	4.56051	33.4684	23.6203
Mvp	NM_080638	17.0722	58.1922	30.8833
Caskin2	NM_080643	3.401	7.27831	3.93647
BC017612	NM_133214	6.09207	16.628	11.9263
Ier3	NM_133662	1.44168	12.4666	2.48811
Ssbp4	NM_133772	1.66572	9.66254	5.34689
Ifi44	NM_133871	0.367288	1.59476	0.967415
Spon2	NM_133903	1.42763	5.38634	7.18336
Cdcp1	NM_133974	0.29593	1.03533	0.701488
Rdh5	NM_134006	11.9908	28.8224	20.3911
Nrm	NM_134122	0.383399	2.02422	1.17428
Syt12	NM_134164	1.73169	6.44573	2.60633

Galnt10	NM_134189	0.520916	1.88698	1.76212
Slc22a27	NM_134256	0.160019	1.12629	2.53157
Hlcs	NM_139145	1.10163	3.86484	2.83188
Tbc1d19	NM_144517	0.541476	1.79317	1.37414
Nmnat3	NM_144533	1.38663	4.19702	4.81406
Klhdc8a	NM_144810	0.417545	1.25667	0.990116
BC021614	NM_144869	19.0378	53.6801	31.8328
Oasl1	NM_145209	2.41002	6.52087	4.99814
Oas2	NM_145227	0.651188	1.8056	1.24983
Acnat2	NM_145368	10.2072	46.0447	29.373
Sectm1a	NM_145373	0.243109	1.95607	1.30377
Slc44a3	NM_145394	1.56295	4.60577	4.8479
Nr1d1	NM_145434	8.58257	19.9541	43.2406
9030617O03Rik	NM_145448	6.68238	13.8538	19.1244
Ifi27l2b	NM_145449	2.91401	53.2457	50.0725
Sdcbp2	NM_145535	0.083145	1.08671	0.805945
Tmem184c	NM_145599	5.54465	11.7818	7.91706
Soat2	NM_146064	12.2619	32.2501	25.1418
Lyplal1	NM_146106	7.15365	15.7685	14.9806
Fam129b	NM_146119	1.04015	6.02839	3.1313
Pbxip1	NM_146131	2.6197	5.5915	4.07265
Cchcr1	NM_146248	0.492633	1.44574	1.0986
Nudt18	NM_153136	2.7988	8.59451	4.43651
Slc36a1	NM_153139	4.50399	11.312	10.5237
Nckap1l	NM_153505	0.353017	1.17319	1.37074
Pilra	NM_153510	0.397819	1.33345	1.60856
Igsf11	NM_170599	6.15527	12.6642	11.2987
Cc2d2a	NM_172274	0.190192	0.625497	0.871919
Rnf43	NM_172448	2.43767	4.81086	7.22184
4930539E08Rik	NM_172450	0.0636975	0.452707	0.191529
Lrp11	NM_172784	0.513402	1.89055	1.24326
Pcyox1l	NM_172832	0.32609	1.76744	0.904758
Pfkfb4	NM_173019	0.145851	0.883382	0.402147
Klhdc7a	NM_173427	3.24799	7.2613	9.75917
Olfm2	NM_173777	0.106019	0.835612	0.760283
Fam83a	NM_173862	0.302284	11.7499	2.83592
Mmgt2	NM_175002	0.481192	2.27732	1.02651
Trib3	NM_175093	1.32499	8.05098	6.78285
Trpm4	NM_175130	0.260016	1.13915	0.961719
Dnaic1	NM_175138	0.0434064	1.92088	0.697389
Mmd2	NM_175217	0.297584	2.48272	6.65961
Sowahb	NM_175270	1.02074	3.60785	2.22579
Nhlrc1	NM_175340	0.261239	1.35584	1.10479
Fam26f	NM_175449	0.437157	2.66798	1.85042
Sptlc3	NM_175467	0	0.585969	1.16352
A2m	NM_175628	0.0333337	16.0872	0.159154
A230050P20Rik	NM_175687	14.8841	40.2117	33.9076

B430306N03Rik	NM_177083	0.206486	0.890366	0.930552
Ccdc66	NM_177111	1.195	2.59391	2.05239
Psd4	NM_177611	0.294255	1.94103	1.51426
E130311K13Rik	NM_177856	2.08634	6.45289	6.0041
Hapln4	NM_177900	1.52204	3.89937	3.50355
Serpina7	NM_177920	21.8755	111.124	74.99
Commd10	NM_178377	2.56458	6.47342	5.70961
Ptrh1	NM_178595	0.394459	2.25057	1.22718
Fbxl21	NM_178674	0.599715	3.75936	3.96106
Sirt5	NM_178848	6.09683	16.8847	15.4936
Pld4	NM_178911	0.8399	3.7415	3.99811
Mtmr11	NM_181409	0.363303	1.17816	0.445021
Ppp1r9a	NM_181595	1.02552	2.34095	2.21783
Gbp6	NM_194336	0.617004	2.96223	2.55244
Them6	NM_198607	1.33959	5.10097	3.70742
Fam211a	NM_198861	0.537035	3.39232	0.596322
Rtn4rl2	NM_199223	0.161963	3.3582	0.210218
Tlr12	NM_205823	2.384	10.5556	12.8465
Dnph1	NM_207161	4.37953	12.2423	12.4193
Ccdc120	NM_207202	0.19831	2.51846	0.840217
D7Ertd715e	NR_015456	0.440282	1.70855	1.27685
Mug-ps1	NR_027619	6.06419	21.1998	19.8784
D930048N14Rik	NR_027958	0.648056	2.08727	2.29919
1810064F22Rik	NR_027981	0.649767	2.08471	1.13588
BC025920	NR_030677	0.101727	0.734091	0.596466
B430212C06Rik	NR_033214	0.108088	0.929329	0.757426
2610507I01Rik	NR_037964	4.38066	8.60699	8.06861
Gm19619	NR_040428	0.573514	2.03378	2.36596
4930556M19Rik	NR_045065	1.36553	3.29038	3.39138
9330102E08Rik	NR_077223	0.073111	0.478885	0.325787
Ttc39aos1	NR_131195	0.230724	2.48922	1.81796

Supplementary Table 3b: Genes down-regulated in sham compared to control (222 genes)

Gene Name	*Gene ID*	*CTL_FPKM*	*SHAM_FPKM*	*VSG_FPKM*
Tmem204	NM_001001183	4.12711	0.860403	1.86892
Zfp36l2	NM_001001806	33.8112	10.4913	18.4461
Hecw2	NM_001001883	0.196159	0.0395712	0.0694782
Syne2	NM_001005510	2.39591	1.31835	1.53107
Slc25a51	NM_001009949	61.2916	14.5847	12.8555
Akr1c19	NM_001013785	60.1524	12.9014	48.1477
Pdp2	NM_001024606	11.1226	3.71435	5.91431
N4bp2	NM_001024917	2.49186	0.785624	1.64054
Gm608	NM_001029889	4.83909	2.49001	3.44808
Acmsd	NM_001033041	10.7375	2.04753	1.5103
Ttc7b	NM_001033213	8.66132	2.99864	4.77528
Slc4a7	NM_001033270	1.83417	0.644781	1.07506

Gm129	NM_001033302	12.1493	3.94335	8.62955
Shb	NM_001033306	38.9788	14.8026	15.9152
Gm826	NM_001033411	5.44257	1.34232	2.02155
Heca	NM_001033432	12.9158	4.86818	5.39219
Gpcpd1	NM_001042672	7.97533	2.6869	2.22299
Cps1	NM_001080809	663.741	112.598	221.732
Cib3	NM_001080812	7.73524	0.142171	0.307985
Lonrf1	NM_001081150	4.61609	1.93035	2.31823
Gpr116	NM_001081178	2.96694	0.399534	1.13986
F830016B08Rik	NM_001101475	6.7297	2.64423	6.90451
Cyp3a59	NM_001105160	9.32061	2.07647	18.9959
Slc16a10	NM_001114332	30.3541	12.78	10.3053
Lpin1	NM_001130412	19.514	0.63867	1.32609
Fam174b	NM_001162532	1.42697	0.260122	0.60513
Ppp1r10	NM_001163818	12.3669	5.64738	4.71501
Syde2	NM_001166064	1.87633	0.685311	1.3191
Zbed6	NM_001166552	4.01647	1.3577	3.96922
BC005561	NM_001166581	1.40997	0.416273	0.829555
Cyp2c50	NM_001167877	12.6357	3.72625	11.2085
Cep85l	NM_001204983	5.25584	1.83922	2.20954
Lcp1	NM_001247984	10.652	2.2796	8.77728
Upp2	NM_001289660	7.39208	0.595162	4.16106
Aqp1	NM_007472	15.7325	3.13974	10.5957
Bmp2	NM_007553	2.92857	0.751931	1.99038
Bmp5	NM_007555	1.71027	0.545839	1.70355
Cry1	NM_007771	5.73112	1.82218	2.21885
Cyp2c29	NM_007815	588.055	31.6565	288.401
Cyp4b1	NM_007823	4.01018	1.36104	2.18733
Dnase1l3	NM_007870	5.71959	1.49681	4.76697
Flt4	NM_008029	2.16957	0.672851	1.55013
Fosl2	NM_008037	6.59436	3.19093	2.09854
G6pc	NM_008061	278.094	50.9839	154.041
Gja4	NM_008120	3.57002	0.655069	1.70485
Gsta2	NM_008182	11.4015	0.65549	3.19342
Hdc	NM_008230	14.4503	3.19232	4.09443
Hspg2	NM_008305	0.882853	0.292609	0.640565
Igfbp3	NM_008343	5.95176	1.54106	2.98221
Kcnb1	NM_008420	0.24734	0.0355987	0.128157
Map3k5	NM_008580	15.8429	6.66469	5.54918
Ppp1r15a	NM_008654	10.8448	4.36158	5.95768
Nos3	NM_008713	1.10797	0.233	0.422402
Dusp8	NM_008748	1.45593	0.341086	0.256204
Cdk18	NM_008795	20.22	10.5207	13.6814
Rrm2	NM_009104	6.08023	1.70293	2.12415
Scd1	NM_009127	1281.15	19.278	45.5797
Serpine2	NM_009255	25.1444	7.0777	6.31106
Thbd	NM_009378	1.46231	0.218891	0.957805

Wee1	NM_009516	5.94449	2.28492	5.02619
Arhgap5	NM_009706	10.9587	5.28366	6.51255
Camk4	NM_009793	0.154467	0.0321407	0.0446878
Cyp2b10	NM_009999	11.1544	0	0.0641658
Cyp2c40	NM_010004	1.82381	0.13598	0.311929
Cd55	NM_010016	2.90087	0.792285	1.99217
Ddx3x	NM_010028	60.4462	25.3624	27.5809
Ctgf	NM_010217	5.05072	1.08291	1.15624
Fkbp5	NM_010220	70.5941	20.7587	14.1836
Flt1	NM_010228	0.557888	0.116252	0.295649
Got1	NM_010324	209.08	43.6552	53.1593
Kdr	NM_010612	2.22553	0.411308	1.47809
Mafb	NM_010658	36.2641	8.45354	19.9447
Lama3	NM_010680	1.05412	0.428679	0.569801
Lipg	NM_010720	7.62194	1.47292	4.09023
Sik1	NM_010831	22.5277	7.43775	7.13119
Nfe2l3	NM_010903	0.40302	0.0234626	0.114044
Oit3	NM_010959	4.97513	1.19797	3.12594
Pltp	NM_011125	29.2081	13.4098	17.5943
Ptp4a1	NM_011200	65.3771	27.3805	21.6651
Ranbp2	NM_011240	11.2872	6.40097	7.79207
Slc22a5	NM_011396	43.0837	17.1205	16.8242
Smarca2	NM_011416	17.9772	7.89107	14.3462
Sox12	NM_011438	1.08618	0.296127	0.560656
Tgfbr3	NM_011578	1.11415	0.446769	0.869743
Tie1	NM_011587	2.42769	0.66648	1.48598
Timp3	NM_011595	1.25802	0.0923087	0.661725
Vipr1	NM_011703	3.2989	1.0881	3.03071
Zfp259	NM_011752	33.9781	13.3134	11.182
Adam23	NM_011780	1.56573	0.429374	1.24455
Car14	NM_011797	20.8404	11.301	19.134
Gadd45g	NM_011817	199.358	39.4876	16.5248
Grem2	NM_011825	8.17691	3.36268	2.7455
Lamc3	NM_011836	0.841581	0.157559	0.124859
Angptl2	NM_011923	2.44603	0.67966	2.50887
Acot1	NM_012006	88.5006	11.8079	26.0392
Kitl	NM_013598	0.833349	0.146621	0.251925
Dusp1	NM_013642	27.8672	8.17442	32.9919
Podxl	NM_013723	0.767106	0.118935	0.460417
Slco1a1	NM_013797	105.803	26.1144	45.6904
Aass	NM_013930	142.363	40.0247	54.6424
Chrna4	NM_015730	0.239016	0.0148415	0.0154976
Sult1d1	NM_016771	109.156	36.9025	40.4334
Nfil3	NM_017373	45.0352	20.8076	11.7577
Slc5a3	NM_017391	0.745684	0.293988	0.383863
Agpat6	NM_018743	63.7865	22.2761	37.6665
Tbc1d8	NM_018775	9.90508	5.20839	4.16207

Calcrl	NM_018782	1.15518	0.394263	1.41247
Plxnc1	NM_018797	1.42445	0.274261	1.11328
Ap3m1	NM_018829	34.4324	13.5177	17.3699
Smad9	NM_019483	4.24425	1.11236	0.639597
Gdf2	NM_019506	4.25817	1.56883	2.95537
Arhgap31	NM_020260	0.622771	0.144055	0.371871
Ehd3	NM_020578	8.34343	2.82495	5.43602
Slc43a3	NM_021398	10.5128	3.71403	6.10466
Shank3	NM_021423	0.782153	0.218324	0.503928
Slc13a2	NM_022411	1.23317	0.0592498	0.173925
Mcam	NM_023061	1.51977	0.418566	0.945291
Sftpa1	NM_023134	1.40914	0.324571	0.629615
Arid5b	NM_023598	13.0091	4.67005	3.95312
Jam2	NM_023844	1.85239	0.404418	1.2597
Clec14a	NM_025809	1.10969	0.331997	1.06121
Acadsb	NM_025826	47.8625	17.7956	26.6857
Bag4	NM_026121	11.1143	5.79086	7.71723
Ankrd33b	NM_026153	2.72892	0.759782	1.43391
Ahctf1	NM_026375	8.49166	3.928	4.41296
Gpihbp1	NM_026730	14.0372	3.63214	9.88167
Mob1b	NM_026735	6.89648	2.21629	4.02253
Esam	NM_027102	5.07272	1.52091	3.26009
Ipmk	NM_027184	20.8033	9.36268	10.3098
Pgm2l1	NM_027629	1.04133	0.339205	0.512799
Cyp2c55	NM_028089	2.29858	0.035197	1.45295
Ddhd2	NM_028102	14.3096	5.98062	6.72657
Rbm33	NM_028234	8.43699	4.05923	5.23175
Gulp1	NM_028450	0.550284	0.0245361	0.0510228
Rasip1	NM_028544	1.62476	0.569833	0.986234
Lonrf3	NM_028894	2.06088	0.718113	1.01032
Cyp2j9	NM_028979	4.95825	2.08415	3.37278
Tmx4	NM_029148	6.02136	2.70876	2.97804
Fam35a	NM_029389	22.6602	7.49775	9.65801
Clec4g	NM_029465	27.36	8.9884	20.0081
Atp11b	NM_029570	12.9525	6.54883	6.82308
Ppp1r3g	NM_029628	12.5304	0.553316	1.44847
Unc5b	NM_029770	1.62042	0.592104	0.935655
Crebrf	NM_029870	6.21404	3.06323	3.29704
Ptprb	NM_029928	2.62646	0.46736	1.37047
Chst15	NM_029935	1.78116	0.387083	1.01399
Adamtsl2	NM_029981	2.88627	0.673246	0.959287
Aacs	NM_030210	26.2179	10.8152	9.29096
Avl9	NM_030235	3.86928	2.03402	2.4177
Slco1a4	NM_030687	10.8128	1.68243	13.2938
Akap12	NM_031185	0.436417	0.07449	0.200592
Krt23	NM_033373	1.5241	0.193975	0.540743
Vps37a	NM_033560	8.50036	4.22757	5.80613

Clec2h	NM_053165	1.14262	0.133785	1.54728
Ugt2b37	NM_053215	3.78651	0.560663	0.922078
Lyve1	NM_053247	3.82058	1.2098	1.01678
Srgap3	NM_080448	0.260068	0.0406786	0.0356026
Elovl6	NM_130450	19.4884	5.0505	5.74107
Eltd1	NM_133222	1.24704	0.314518	1.02875
Fbxo31	NM_133765	47.3708	18.5812	10.675
Gpr110	NM_133776	1.73988	0.0412203	0.0683312
Ugt2b38	NM_133894	16.3866	1.2783	1.16053
N4bp2l1	NM_133898	28.073	13.1717	25.7316
Acacb	NM_133904	17.8002	6.00617	4.743
Ces2a	NM_133960	229.129	18.1563	85.7101
Gstt3	NM_133994	24.8857	7.22079	17.4666
Acot3	NM_134246	15.8951	2.92186	23.2769
Sdpr	NM_138741	2.40937	0.558086	1.44848
Mib1	NM_144860	5.24337	1.94846	3.0186
Rprd1a	NM_144861	6.84122	3.47521	3.25398
L2hgdh	NM_145443	18.9279	8.89789	15.1048
Farp2	NM_145519	4.93767	2.09728	3.12587
Ces2c	NM_145603	11.7529	3.12437	7.39539
Pank3	NM_145962	13.8144	6.88214	7.73512
Rhbdd2	NM_146002	9.71181	4.74447	5.7409
Trhde	NM_146241	0.304302	0.0497978	0.251234
Abca6	NM_147218	32.7737	13.0983	23.292
Ugt2b1	NM_152811	248.383	42.0904	87.2829
Mmrn2	NM_153127	1.14249	0.366148	0.823641
Dcaf10	NM_153167	5.69817	2.94158	2.91476
Gimap6	NM_153175	3.70983	1.18946	3.1912
Csrnp1	NM_153287	7.32536	3.15443	3.41489
Cmtm4	NM_153582	10.9917	5.68375	6.34924
Polr2b	NM_153798	16.1697	8.50705	11.7661
Dpyd	NM_170778	115.194	54.0686	79.9426
Slc25a32	NM_172402	9.08663	3.8735	4.94898
Zfp871	NM_172458	11.0404	5.78767	7.96941
She	NM_172530	0.395893	0.0741399	0.242726
Fbf1	NM_172571	22.0108	7.35223	3.73463
Lrp4	NM_172668	4.95796	2.65146	4.31562
Ttll8	NM_172818	2.60384	0.349121	0.237064
Vps13a	NM_173028	2.51037	0.829586	1.28755
Colec10	NM_173422	1.75799	0.645813	2.14698
Ppip5k2	NM_173760	15.5008	7.27576	7.58829
Crybg3	NM_174848	3.74691	2.51696	2.58048
Tctex1d4	NM_175030	1.26355	0.174863	0.0706104
Slc38a2	NM_175121	94.6171	27.2138	27.6415
Srd5a1	NM_175283	44.9065	15.062	19.5244
E130012A19Rik	NM_175332	3.7474	0.338422	0.714755
Gpd1l	NM_175380	8.50852	4.3297	7.38802

Slc35g1	NM_175507	14.0703	5.36259	7.17345
Clec1a	NM_175526	0.377068	0.0427019	0.16323
Wnk4	NM_175638	1.1679	0.132624	0.312315
Glt1d1	NM_177005	20.3036	7.63865	14.1006
Lrrc58	NM_177093	13.663	6.92255	6.80059
Lmbrd2	NM_177178	4.29381	1.40418	3.19029
Pptc7	NM_177242	5.56258	2.02549	2.93257
Arhgef15	NM_177566	1.49123	0.376653	1.03662
Arhgef37	NM_177828	1.99601	0.602546	1.24006
Col14a1	NM_181277	3.2388	0.997466	3.3461
4931429L15Rik	NM_183104	0.37378	0	0.04853
Fam107a	NM_183187	1.36825	0.0373483	0.0365341
Usp2	NM_198091	21.6524	7.46362	12.256
Stard8	NM_199018	1.40296	0.536531	0.858115
Ugt1a5	NM_201643	20.0479	3.51528	2.99855
Ugt1a9	NM_201644	23.172	3.0444	8.50645
Btbd19	NR_024078	6.12463	0.726984	0.55582
Rian	NR_028261	0.658899	0.111605	0.193257
1500017E21Rik	NR_033510	15.1399	3.59155	13.2343
Gm19522	NR_040402	2.62893	0.517535	0.62509
9430037G07Rik	NR_040766	3.09675	0.615018	0.782263
Gm16551	NR_045284	3.84229	1.27321	1.70914

Supplementary Table 4: The Primers for RT-PCR.

Gene	Sequence of forward and reverse primers (5'-3')	GeneBank Accession NO.
M36B4	GCCCTGCACTCTCGCTTTCT	NM_007475
	CAACTGGGCACCGAGGCAACAGTTG	
Cyp2c29	TTTTTCAGCCATTGGAAAGC	NM_007815
	TGGGCTCAAAGCCTACTGTC	
Cyp3a11	GGGGGACAGCAAAGCTCTAT	NM_007818
	TTCTGTCTTCACAAACCGGC	
Cyp2c55	TCCCTGGGAGATAATTGATGA	NM_028089
	CTTGATGGAGAGACTCAATGAAAA	
